# pLM-SAV: a Δ-embedding approach for predicting pathogenic single amino acid variants

**DOI:** 10.1093/bioadv/vbag195

**Published:** 2026-07-15

**Authors:** Orsolya Gereben, Hedvig Tordai, Lana Khamisi, Erda Qorri, Tamás Hegedűs

**Affiliations:** Department of Biophysics and Radiation Biology, Semmelweis University, Budapest 1094, Hungary; Department of Biophysics and Radiation Biology, Semmelweis University, Budapest 1094, Hungary; Department of Biophysics and Radiation Biology, Semmelweis University, Budapest 1094, Hungary; Mutagenesis and Carcinogenesis Research Group, HCEMM-HUN-REN BRC, Szeged 6728, Hungary; Doctoral School of Biology, Faculty of Natural Sciences, University of Szeged, Szeged 6720, Hungary; Department of Biophysics and Radiation Biology, Semmelweis University, Budapest 1094, Hungary; Biophysical Virology Research Group, HUN-REN-SU, Budapest 1052, Hungary

## Abstract

**Motivation:**

Predicting whether single amino acid variants (SAVs) in proteins lead to pathogenic outcomes is a critical challenge in molecular biology and precision medicine. Experimental determination of all possible mutation effects is infeasible, and while state-of-the-art tools such as AlphaMissense show promise, their diagnostic performance is insufficient and they are often difficult to run locally.

**Results:**

We developed pLM-SAV, a simple yet effective predictor that leverages protein language models (pLMs). Δ-embeddings, computed as the difference between wild-type and mutant sequence embeddings, are used as input for a convolutional neural network. We trained our model on a well-characterized, labelled set of Eff10k and evaluated it on a non-homologous subset of ClinVar data. This approach performs exceptionally well on the Eff10k test folds and reasonably on ClinVar test sets. On ambiguity-defined subsets, pLM-SAV provides complementary predictions to AlphaMissense and REVEL, although these methods retain higher overall performance on broad ClinVar benchmarks. Our results show that a compact predictor trained on labelled variant-effect data can provide useful predictive performance across multiple benchmarks. Unlike previous methods such as VESPA, pLM-SAV uses no handcrafted features or substitution matrices, relying solely on pLM-derived representations. Δ-embedding-based features may provide useful additional signals for future mutation-effect predictors or integrative models.

**Availability and implementation:**

The code is available at https://doi.org/10.5281/zenodo.15502498.

## 1 Introduction

Understanding the functional consequences of single amino acid variants (SAVs) is a major goal in molecular biology, human genetics, and precision medicine (Lek *et al.* 2016, Karczewski *et al.* 2020). SAVs can alter protein structure, dynamics, interactions, or stability, and are often associated with human disease (Arnold *et al.* 2009, Sattar *et al.* 2020). As high-throughput sequencing continues to uncover vast numbers of novel variants, which include many variants of uncertain significance (VUS), reliable in silico tools are needed to predict whether a given amino acid substitution is likely to be pathogenic or benign. However, experimental validation of all possible variants is impractical, and often impossible, especially for large protein families or non-model organisms (Drozda *et al.* 2018, Findlay *et al.* 2018).

Over the past decade, many computational methods have been developed to estimate the functional impact of SAVs. Among the most widely used are rule-based and supervised learning models that combine evolutionary conservation with structural, biophysical, or annotation-derived features. An important early method is SNAP2 (Hecht *et al.* 2015). SNAP2 is a supervised predictor trained using 10-fold cross-validation on the Eff10k dataset. It combines a wide range of features, including evolutionary and structural data, such as sequence alignments, secondary structure, solvent accessibility, and biophysical properties. It also integrates curated annotations and functional signals from resources like SWISS-Prot, Pfam, PROSITE, and AAindex, making it a feature-rich and biologically informed model.

REVEL (Rare Exome Variant Ensemble Learner) (Ioannidis *et al.* 2016) is a powerful ensemble predictor combining multiple tools to prioritize likely pathogenic variants, and has been widely adopted in clinical genomics pipelines. DeepMind’s AlphaMissense (Cheng *et al.* 2023) provides predictions for 71 million single-nucleotide missense variants and a superset of 216 million possible amino acid substitutions. It builds on the AlphaFold2 framework, using structure-based features to train a deep learning model. AlphaMissense has shown strong performance on standard benchmarks such as ClinVar and ProteinGym, but its diagnostic utility is still limited. Moreover, it is restricted to human proteins, and its models are not easily deployed or retrained locally.

Among the more recent generation of embedding-based predictors is VESPA, developed by Marquet *et al.* (2022). VESPA infers conservation using protein language model (pLM) embeddings and supplements this information with BLOSUM substitution scores and amino acid substitution probabilities. Importantly, VESPA and its variant VESPAl were trained on the Eff10k dataset using a methodology similar to ours in this study, making them highly relevant for direct comparison. VariPred (Lin *et al.* 2024) among other features uses the wild type and mutant embeddings of the ESM-1b language model at the mutation position and concatenates them to create input features for SAV prediction. Several methods have been developed that exploit pLMs in diverse ways to predict SAV effects (Brandes *et al.* 2023, Yan *et al.* 2024).

The emergence of pLMs has transformed computational biology by enabling high-quality sequence embeddings learned from massive databases of unlabelled protein sequences. These models, such as ProtT5 (Elnaggar *et al.* 2022) and Ankh (Elnaggar *et al.* 2023) capture rich contextual and evolutionary information without the need for multiple sequence alignments. Protein embeddings generated by pLMs have already shown promise for downstream tasks, including structure prediction, function annotation, and variant effect estimation (Heinzinger and Rost 2025, Wang *et al.* 2025).

Delta embeddings calculated from sequence-wise average embeddings (Gong *et al.* 2023) of the mutant and the wild type protein and full sequence wild type and mutant embedding difference (Savojardo *et al.* 2024) were used previously for thermostability predictions, but they have not applied in SAV pathogenicity prediction previously.

Here, we explore whether Δ-embeddings, the difference between the embedding vectors of wild-type and mutant protein sequences, provide a mutation-centred representation that is useful for SAV effect prediction. Although pLM embeddings encode sequence context, it remains unclear to what extent their difference captures information relevant to variant classification and how it compares with alternative ways of combining wild-type and mutant embeddings. To address this, we developed pLM-SAV, a lightweight convolutional neural network (CNN) model trained on delta-embeddings derived from either ProtT5 or Ankh (Elnaggar *et al.* 2022, 2023). The model was trained on a clustered, non-redundant subset of the Eff10k dataset (Hecht *et al.* 2015, Marquet *et al.* 2022) and evaluated on ClinVar proteins with no sequence similarity to the training data (see Section 2), ensuring robust generalization. Additionally, we assessed pLM-SAV’s performance on ambiguous cases predicted by AlphaMissense and REVEL, and compared it to other predictors on the ProteinGym benchmark.

## 2 Methods

### 2.1 Datasets

Eff10k, a dataset originally developed in the Rost laboratory (https://www.rostlab.org) for the SNAP2 and VESPA predictors (Hecht *et al.* 2015, Marquet *et al.* 2022), was obtained from the authors along with their predefined fold split. The set was combined from three sources: PMD (the Protein Mutant Database) (Kawabata *et al.* 1999), protein-specific studies (Bairoch and Apweiler 2000, Dimmer *et al.* 2012), and disease variants from OMIM (Online Mendelian Inheritance in Men) (Hamosh *et al.* 2005, Capriotti *et al.* 2006), as detailed in Hecht *et al.* (2015). The authors clustered protein sequences using PSI-BLAST with an *E*-value threshold of <1e–3 to group homologous proteins. The cluster sizes varied greatly (1–1941 members) (Hecht *et al.* 2015). These clusters were then randomly assigned to 10 folds, resulting in one disproportionately large fold (Fold 0, containing 44 024 variants) and nine approximately equal-sized folds. This clustering and folding strategy ensured that no homologous sequences were shared across folds, thereby preserving the integrity of cross-validation and preventing data leakage. We adopted this fold assignment unchanged for comparability with SNAP2 and VESPA. The full dataset contains 100 737 binary SAV-effect annotations from 9594 proteins, of which 99 926 were used in our analysis (39 299 classified as neutral and 60 627 labelled as effect) with an effect-to-neutral ratio of ∼1.5.

PMD4k is a subset of the Eff10k dataset derived from the Protein Mutant Database (PMD) (Kawabata *et al.* 1999). It has been used for testing in previous SAV effect prediction methods, including SNAP2 (Hecht *et al.* 2015) and VESPA (Marquet *et al.* 2022). The dataset comprises 51 266 variants, including 13 497 classified as neutral and 37 769 as effect. It can be further divided into 676 human proteins with 2788 neutral and 6863 effect variants, and 3343 non-human proteins with 10 709 neutral and 30 906 effect variants. To prevent data leakage, we followed the approach of Rost and colleagues (Hecht *et al.* 2015, Marquet *et al.* 2022) and evaluated predictions only for PMD4k variants in the test fold of each model. Since PMD4k is a subset of Eff10k, the PMD variants were assigned to the same folds as their parent sequences, resulting in 10 smaller folds nested within the Eff10k folds. This ensured that sequence-similar proteins were not shared across training, validation, and test sets.

ClinVar entries containing single amino acid benign or pathogenic mutations with at least one star were used for additional testing downloaded in November 2023. Similarly, for the ClinVar dataset, we applied PSI-BLAST filtering (with an *E*-value threshold of <1e−3) to remove proteins in the test set that were sequence-similar to those in the training and validation folds, ensuring strict independence. This approach led to slightly different test subsets for our training methods ‘A’ and ‘B’ (see method description below) and ensured a strict and robust evaluation. ClinVar entries containing benign and pathogenic missense mutations with at least one star were used for additional testing. For method ‘A’, this resulted in the CV-A set, containing 17 677 variants (15 867 benign and 1810 pathogenic). For method ‘B,’ the CV-B set included 83 642 variants (58 159 benign and 25 483 pathogenic).

To enable a direct comparison with VariPred (Lin *et al.* 2024), the CV-B dataset was filtered to retain only proteins that were also present in the VariPred test set. The resulting CVari dataset contained 29 981 variants and was used to compare the performance of pLM-SAV (method ‘B’ with Ankh) against VariPred. The VariPred predictions on CVari set can be found in pLM-SAV_SUPPData.xlsx and on Zenodo.

Further comparisons were conducted with AlphaMissense (AM) (Cheng *et al.* 2023) and REVEL (Ioannidis *et al.* 2016). AlphaMissense data were retrieved from our relational PostgreSQL database (Tordai *et al.* 2024), while REVEL data were downloaded from REVEL’s official site (https://sites.google.com/site/revelgenomics/downloads). REVEL variants were identified using Ensembl transcript IDs and GRCh38 positions, mapped with Ensembl EMBL files (version GRCh38.113, https://ftp.ensembl.org/pub/release-113/embl/homo_sapiens), utilizing custom Python scripts. Variants common to AM, REVEL, and either the CV-A or CV-B test sets were selected, resulting in the CCV-A set with 11 144 variants (1131 benign and 10 013 pathogenic) and the CCV-B set with 46 475 variants (33 217 benign and 13 258 pathogenic).

To further assess pLM-SAV’s reliability, we examined different kinds of method-specific ambiguous sets. Variants common between AM’s ambiguous category and any kind of REVEL were selected for evaluation, focusing on cases where AM struggled. Since score calculation required true labels, only ambiguous AM variants with REVEL predictions and reliable ClinVar annotations were included, forming the AMambRCV set with 5503 variants from 2069 proteins. Similarly, RambAMCV, set with 11 007 variants from 2229 proteins, contains the ambiguous REVEL variants with any kind of AM predictions and ClinVar labels. Their intersection, the AMambRambCV set, contained 1104 variants from 737 proteins. The largest ambiguous AM set with ClinVar labels is the AMambCV set with 6010 variants from 2229 proteins, while the largest ambiguous REVEL set with ClinVar labels is the RambCV set with 12 269 variants from 3169 proteins. These set were not independent of our training and validation sets.

Variants with REVEL < 0.28 were considered likely benign, those with REVEL > 0.48 as likely pathogenic, and remaining variants were considered ambiguous. The thresholds of 〖th〗_b^R = 0.28 and 〖th〗_p^R = 0.48 reflect a trade-off between sensitivity and selectivity, with one typically around 75% and the other around 88%, depending on which end of the threshold is considered. For AM, the thresholds in Cheng *et al.* (2023) were used 〖(th〗_b^AM = 0.34, 〖th〗_p^AM= 0.564).

The division between ambiguous or certain predictions based on either AM or REVEL scores was performed for the independent CCV-A and CCV-B sets as well resulting in 581 ambiguous variants for CCV-A and 2676 for CCV-B according to the AM split and 899 ambiguous variants for CCV-A and 5404 for CCV-B according to the REVEL split.

To perform the evaluation of either the AM or REVEL ambiguous sets, these predictions were converted into benign or pathogenic based on the mean of the respective thb–thp threshold ranges, resulting in 0.38 for REVEL and 0.452 for AM. Additionally, we compared AlphaMissense predictions using the multiplexed assays of variant effect (MAVE), also known as Deep Mutational Scanning (DMS), from the ProteinGym benchmark (version 0.1) (Notin *et al.* 2022). This benchmark consists of 1.5 million variants derived from 87 assays covering 72 unique proteins.

### 2.2 Training strategy

Training was conducted on the Eff10k dataset using a nested k-fold cross-validation framework, incorporating separate validation and test sets, to rigorously assess predictor performance while minimizing overfitting and data leakage. In method ‘A’, all 10-folds were utilized (*k* = 10), whereas in method ‘B’ only folds 1 to 9 were used (*k* = 9). Fold 0 was excluded in method ‘B’ due to its disproportionately large size, approximately seven times larger than the average of the other folds in terms of both mutations and proteins. To ensure strict independence, the test set contained no proteins with sequentially similar to those in the training and validation folds, to prevent data leakage.

Training was optimized using BCEWithLogitsLoss (binary cross-entropy loss combined with a sigmoid layer for numerical stability) as implemented in PyTorch (Paszke *et al.* 2019). To account for varying fold sizes, weighting was applied. Early stopping was implemented by monitoring the difference between training and validation losses to prevent overfitting. The optimal probability threshold for binary classification was determined by evaluating the MCC (Matthews correlation coefficient) scores across thresholds from 0.1 to 0.9 in increments of 0.01 on the validation set and selecting the threshold that produced the highest respective score.

In each iteration of k-fold cross-validation, the training set was merged from k-2 folds, the validation set consisted of one-fold for tuning model parameters and assessing performance, and a separate fold was held out as the test set to evaluate generalizability. This resulted in 90 models for method A and 72 for method B, with the best model selected per fold for evaluation. The thresholds for the best models determined on the validation set are given in Table S1, available as supplementary data at *Bioinformatics Advances* online. This nested structure ensured that every fold was used as a test set exactly once throughout the full cross-validation cycle. All reported training results represent averages across the best models, as selecting a single overall best model for a training cycle could lead to overfitting. For method ‘B’, Fold 0 combined with the corresponding test fold was also used as a hybrid prediction.

### 2.3 CNN training with delta embeddings

We evaluated both dense neural networks with varying window sizes and several convolutional architectures (Table S2, available as supplementary data at *Bioinformatics Advances* online). After hyperparameter tuning, a compact CNN (CNN1c) with narrow layers performed best, providing efficient training and fast inference. The full pipeline and CNN1c architecture are shown in Fig. S1, available as supplementary data at *Bioinformatics Advances* online.

For each protein variant, embeddings were generated for the full amino acid sequence of both the wild-type and mutant proteins using ProtT5-XL-U50 (referred to as ProtT5) and the Ankh PLM (Elnaggar *et al.* 2022, 2023). See the model parameters in Table S3, available as supplementary data at *Bioinformatics Advances* online. No additional fine-tuning was performed, as this did not appear to improve the quality of the SAV pathogenicity predictions, but it considerably increased the training time (Radjasandirane *et al.* 2025). The embedding vector of the mutant sequence was subtracted from that of the wild-type sequence on a per-residue basis (Δembeddingi = embeddingWT, i − embeddingmut, i). The resulting feature array for each variant consisted of 7 × Δembedding, with 7 × 1024 dimensions for ProtT5 and 7 × 1536 for Ankh. These 7 × Δembedding arrays were then used as input to a convolutional neural network (CNN) to classify each variant as neutral or effect for the training set (and benign or pathogenic for ClinVar, AM, and REVEL).

### 2.4 Performance metrics used

The description of the performance metrics used and the additional methods are described in Table S4 and Method S1, available as supplementary data at *Bioinformatics Advances* online. Briefly, the mean and sample standard deviation of AUC and MCC were determined from the best 10 and 9 pLM-SAV models for method ‘A’ and method ‘B’, respectively, and bootstrapping (*n* = 1000) was used in the case of AlphaMissense and REVEL data.

## 3 Results

### 3.1 Single amino acid substitutions induce local changes in pLM embedding space

**Figure 1:**
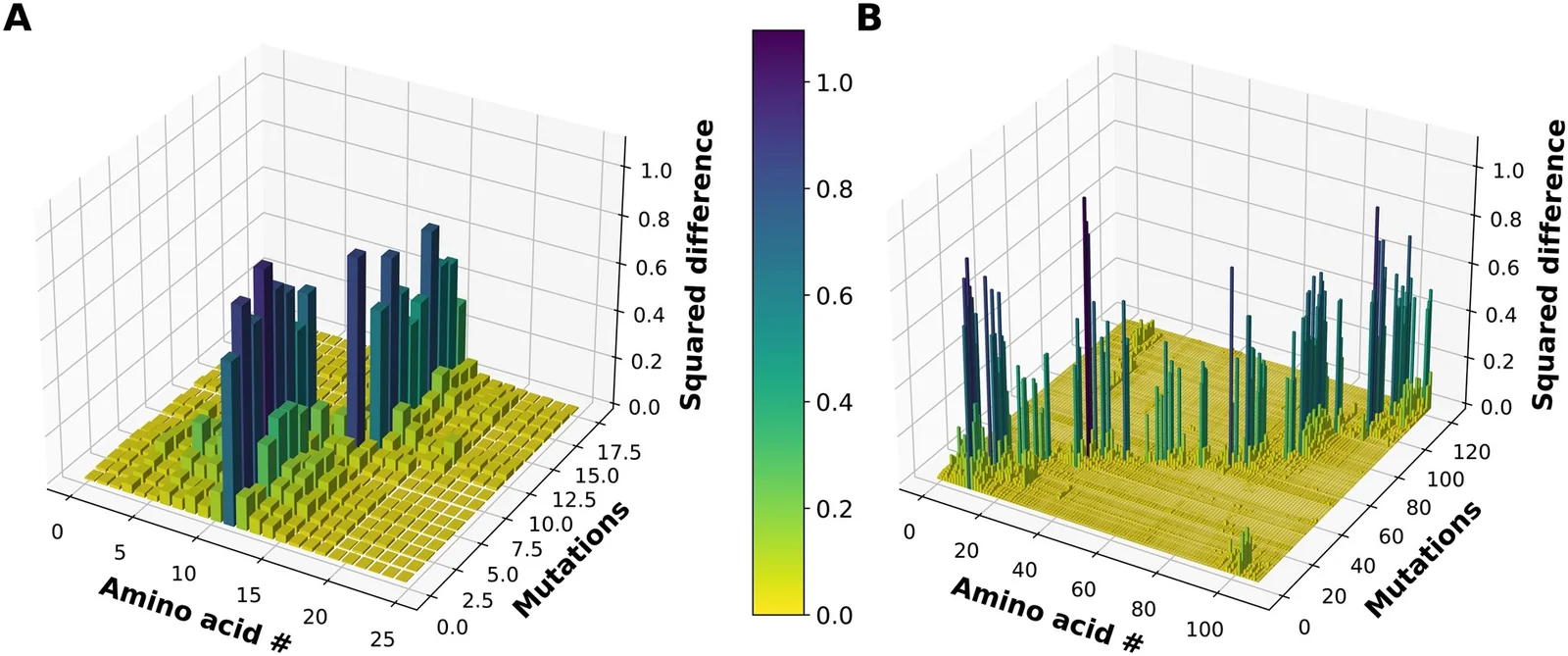
Δ-embeddings reveal local representation changes beyond the mutation site. Squared differences between wild-type and mutant embeddings are shown for Cytochrome c isoform 1 (UniProt: P00044). The embedding difference is largest at the mutation site but is also detectable at neighbouring positions, indicating that the sequence representation changes locally beyond the altered residue. These patterns highlight the suitability of convolutional neural networks (CNNs) for capturing and interpreting such localized embedding differences. (A) Squared differences across the full sequence. (B) A selected region with specific mutations is shown to enhance visibility and illustrate the propagation and decay of the signals.

We first examined whether single amino acid substitutions induce local changes in PLM embedding space that could be used for variant classification. To capture the representational difference of a mutation, the embedding vector of the mutant sequence was subtracted from that of the wild-type sequence on a per-residue basis (Δembeddingi = embeddingWT, i − embeddingmut, i). Notably, while the difference in embeddings was usually most pronounced at the mutation site, significant variations were also observed in the surrounding residues within the selected window. After evaluating different window sizes (Table S2, available as supplementary data at *Bioinformatics Advances* online), a seven-residue window comprising three residues before and three residues after the mutation was selected to best represent the localized difference between the two sequences (Fig. 1). These local representation changes contain predictive information, although their aggregated magnitude alone does not specifically distinguish effect from neutral variants (Table S5 and Figs S2 and S3, available as supplementary data at *Bioinformatics Advances* online).

### 3.2 Performance of Δ-embedding-based predictors

To evaluate predictive performance, we trained and tested models using the Eff10k dataset (Hecht *et al.* 2015), which was partitioned into 10 non-homologous folds to ensure that sequence-similar proteins did not overlap between training, validation, and test sets. For each experiment, one-fold was held out for testing, while the remaining folds were used for model training and validation. We benchmarked two PLMs as embedding sources, ProtT5 (Elnaggar *et al.* 2022) and Ankh (Elnaggar *et al.* 2023), and applied two alternative training strategies: method A, in which the full Eff10k dataset (all 10-folds) was used, with models trained on eight combined training folds, guided by a validation fold, and evaluated on a held-out test fold; and method B, in which the substantially larger fold 0 was excluded and only folds 1–9 were used, with seven combined training folds, a validation fold, and evaluation on a held-out test fold (see Section 2). In the latter case, we also evaluated a merged set of the test fold and fold 0 (Fold i + 0).

**Figure 2:**
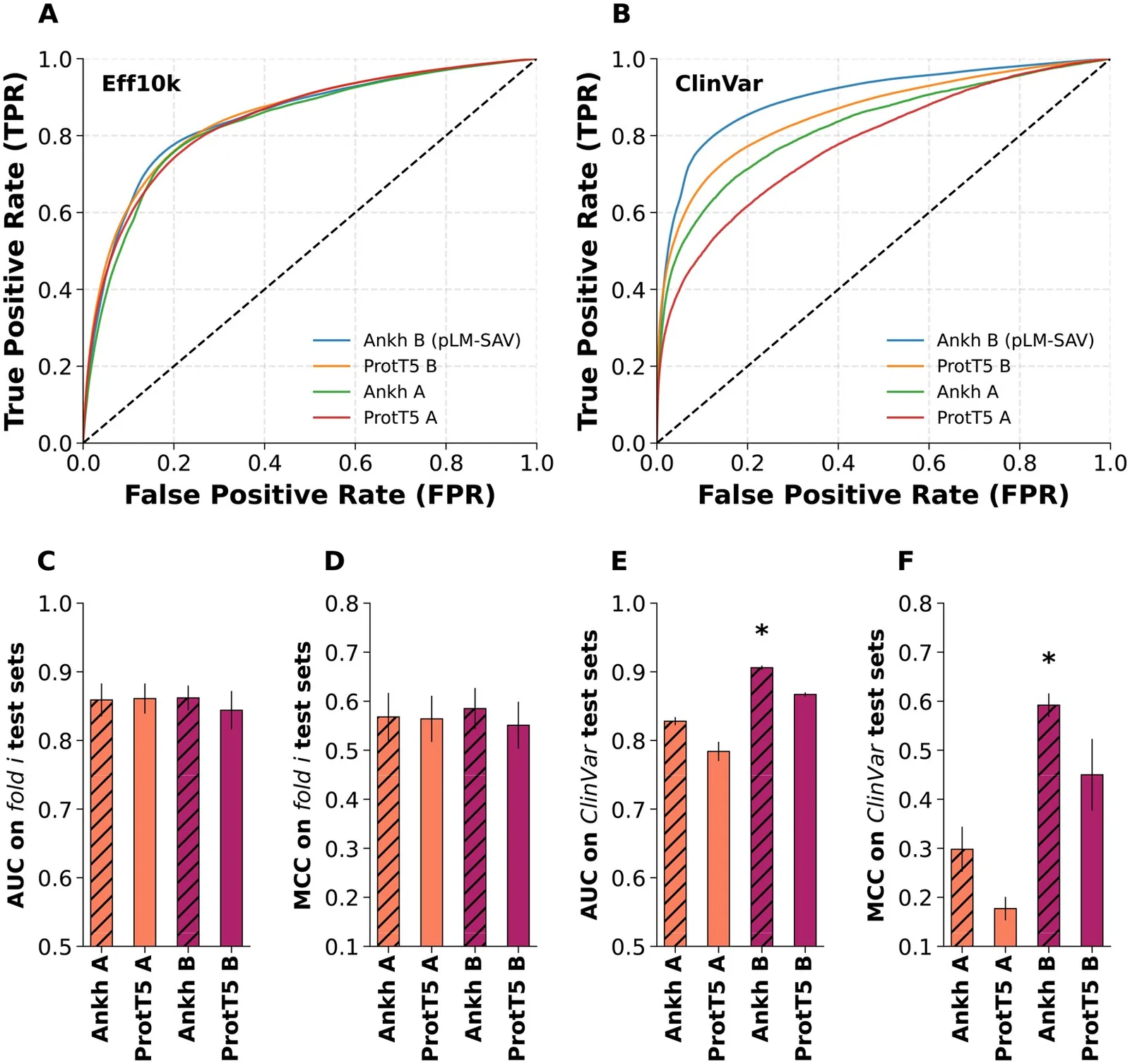
Performance of pLM-SAV using Δ-embeddings on Eff10k and ClinVar test sets. ROC curves are shown for the Eff10k test sets (A) and the ClinVar-derived test sets (B). Corresponding AUC and MCC values are summarized for Eff10k (C and D) and ClinVar (E and F). Models were trained using method A (orange) and method B (raspberry). Embeddings were generated using Ankh (striped bars) and ProtT5 (solid bars). All sets significantly differ (*P* < .05) from the Ankh B set marked with an asterisk on panels E and F. Exact *P-*value are reported in Table S6, available as supplementary data at *Bioinformatics Advances* online.

The average performance metrics of the best convolutional neural network (CNN) models for each training method are presented in Fig. 2, Fig. S4, and Table S6, available as supplementary data at *Bioinformatics Advances* online. In all cases, these metrics exceeded their respective usually used statistical significance thresholds, such as 0.8 and 0.5 for AUC and MCC, respectively. Among all variations, method ‘B’ and the Ankh combination yielded the best performance. Additionally, method ‘B’ allowed for independent predictions on Eff10k Fold 0, in combination with the respective test folds (Fig. S4A and B and Table S6, available as supplementary data at *Bioinformatics Advances* online). The combined predictions yielded lower scores compared to the test fold predictions and method ‘B’ outperformed method ‘A’ overall. These observations suggest that fold 0 is inherently more difficult to predict than the other folds.

Analysis of the Eff10k dataset revealed that effect variants generally exhibit higher summed squared differences within the 7-amino acid Δ-embedding window compared to neutral variants (Fig. S3A). When further separating both classes into true and false predictions by the ‘A’ and ‘B’ Ankh models (Fig. S3B and C, available as supplementary data at *Bioinformatics Advances* online), a consistent tendency emerged: correctly classified variants (true positives/negatives) align with the expected distribution of larger (true positive) or smaller (true negative) differences, whereas misclassified variants (false positives/negatives) often deviate from this pattern. These results indicate that the magnitude of the wild-type–mutant embedding difference contributes to the pLM-SAV input signal. However, the distributions overlap substantially between effect and neutral variants, and therefore no scalar Δ-embedding-magnitude threshold can reliably separate the two classes. The classifier instead uses the complete signed, multidimensional pattern across the local seven-residue window.

To further assess the generalization performance of our models, predictions were made on the two ClinVar datasets, CV-A and CV-B test sets, to evaluate method ‘A’ and method ‘B’, respectively. In order to directly compare the two methods on the same data, CV-A was also predicted using method ‘B’ (Fig. S4C and D and Table S6, available as supplementary data at *Bioinformatics Advances* online). We created these test sets to contain only proteins that are entirely independent of the training and validation sets, without sequence similarity to any proteins in these sets (see Section 2). Among all tested models, only the method ‘B’/Ankh combination produced statistically significant results across all metrics in ensemble predictions (Fig. 2, Fig. S4C and D and Table S6, available as supplementary data at *Bioinformatics Advances* online). The Receiver Operating Characteristics (ROC) and precision-recall (PR) curves including additional data sets as well (Fig. S4, available as supplementary data at *Bioinformatics Advances* online) also support this finding. In every case, the Ankh model outperformed ProtT5, confirming its superior ability to capture information-rich sequence representations. Importantly, the superior performance of method ‘B’ suggests that Eff10k Fold 0 negatively impacts not only predictions but also the training process.

### 3.3 Comparison with a concatenation-based pLM method

There are several possible ways to combine wild-type and mutant embeddings for variant prediction. We therefore compared pLM-SAV with the external concatenation-based VariPred method; however, this comparison is not a controlled representation ablation because the training data, classifier architectures, and thresholding strategy differ. To assess the impact of representation choice, we compared pLM-SAV to a concatenation-based approach implemented in VariPred (Lin *et al.* 2024), where embeddings of the wild-type and mutant residues are appended and interpreted by a shallow classifier.

For a fair comparison, we evaluated both methods on the subset of variants common to the VariPred test set and our independent test set (CVari, see Section 2). It is important to note that the training datasets differ substantially, as VariPred is trained on ClinVar-derived data. On this common test set, pLM-SAV (model B, Ankh) and VariPred achieved AUC values of 0.910 and 0.880, respectively. In contrast, VariPred showed a higher MCC (0.756) compared to pLM-SAV (0.628). This discrepancy between ranking-based (AUC) and threshold-dependent (MCC) metrics may partly reflect differences in score distributions and threshold selection. In particular, VariPred applies a relatively low classification threshold (0.2), optimized on its validation data, which may favour MCC under imbalanced conditions.

### 3.4 Comparison of pLM-SAV to SAV effect predictors

**Figure 3:**
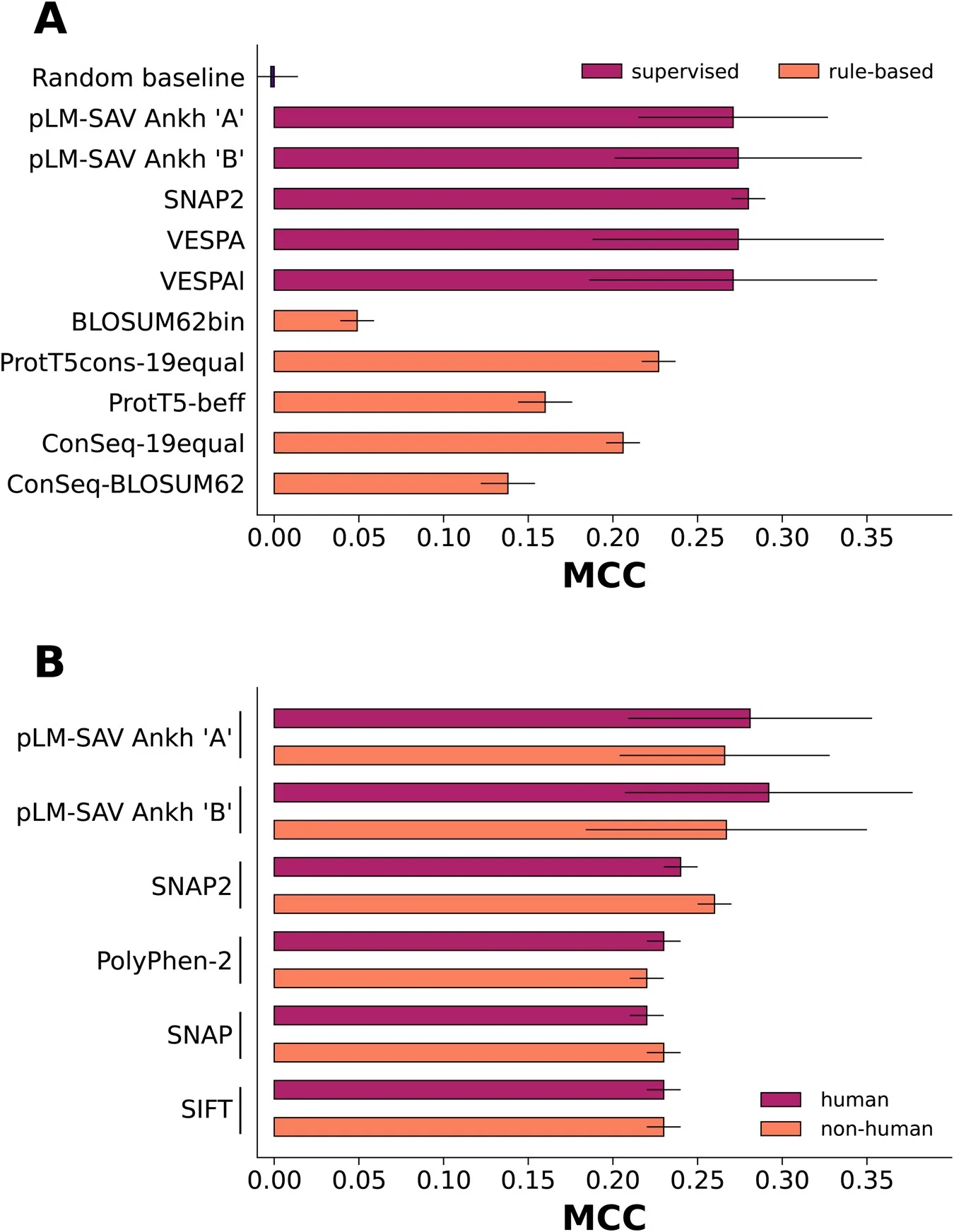
pLM-SAV performance is comparable to supervised and rule-based methods. Mean MCC values and standard errors for PMD4k (A) and the human versus non-human split of PMD4k (B) datasets were collected from published sources, as detailed in Section 2 and the main text. pLM-SAV Ankh ‘A’ and ‘B’ were not statistically different at *P* < .05 level. Difference compared to model pLM-SAV Ankh ‘B’ were tested according to the method given in Section 2: Performance metrics, exact *P-*value can be found in Tables S7 and S8, available as supplementary data at *Bioinformatics Advances* online.

To evaluate the performance of our approach, we compared it to supervised and rule-based methods. We note that our evaluation setting mirrors that of SNAP2 and VESPA, as we obtained the folds from the authors (Hecht *et al.* 2015, Marquet *et al.* 2022), allowing direct comparison. These methods were assessed on the PMD4k set, which is a subset of Eff10k. Therefore, we followed a careful procedure to ensure that predictions were only made for PMD4k proteins that appeared in the independent test fold, mirroring the methodology used in previous studies. No single method consistently outperformed all others across every metric (Fig. 3A, Fig. S5, and Table S7, available as supplementary data at *Bioinformatics Advances* online). SNAP2 achieved the highest MCC score of 0.280, while the pLM-SAV Ankh models performed nearly as well, with scores of 0.274 and 0.271, also comparable to VESPA and its variant, VESPAl. For MCC, the supervised models (pLM-SAV and VESPA) outperformed the rule-based methods. In contrast, for accuracy, the rule-based ConSeq-19-equal method, which categorizes all non-native variants at conserved positions as having an effect, achieved the highest score of 0.715 (Table S7, available as supplementary data at *Bioinformatics Advances* online). These results show that a pLM-SAV model using Δ-embedding inputs can achieve performance competitive with several more feature-rich SAV-effect predictors.

To further assess performance, we evaluated predictions on PMD4k, split into human and non-human proteins, allowing a direct comparison to SNAP2’s development study (Hecht *et al.* 2015). This also allowed additional comparisons made with the widely used methods PolyPhen-2 (Adzhubei *et al.* 2010) and SIFT (Ng and Henikoff 2003). As before, predictions were made only for independent test fold proteins, ensuring a fair evaluation. For human variants, the pLM-SAV ‘B’ Ankh model achieved the best scores in both MCC (0.292) and F1neutral (0.497) (Fig. 3B and Table S8, available as supplementary data at *Bioinformatics Advances* online). For non-human variants, pLM-SAV models consistently achieved the highest scores. However, as with previous comparisons, no single method emerged as the best across all metrics.

Importantly, the mean values and standard deviations for methods other than pLM-SAV in case of PMD4k were obtained from related publications (Hecht *et al.* 2015, Marquet *et al.* 2022); therefore, statistical significance between these methods and ours could not be evaluated. ROC and PR plots were generated for our models and support the superiority of method ‘B’ over ‘A’ (Fig. S5, available as supplementary data at *Bioinformatics Advances* online).

### 3.5 pLM-SAV improves prediction of ambiguous AlphaMissense and REVEL variants

**Figure 4:**
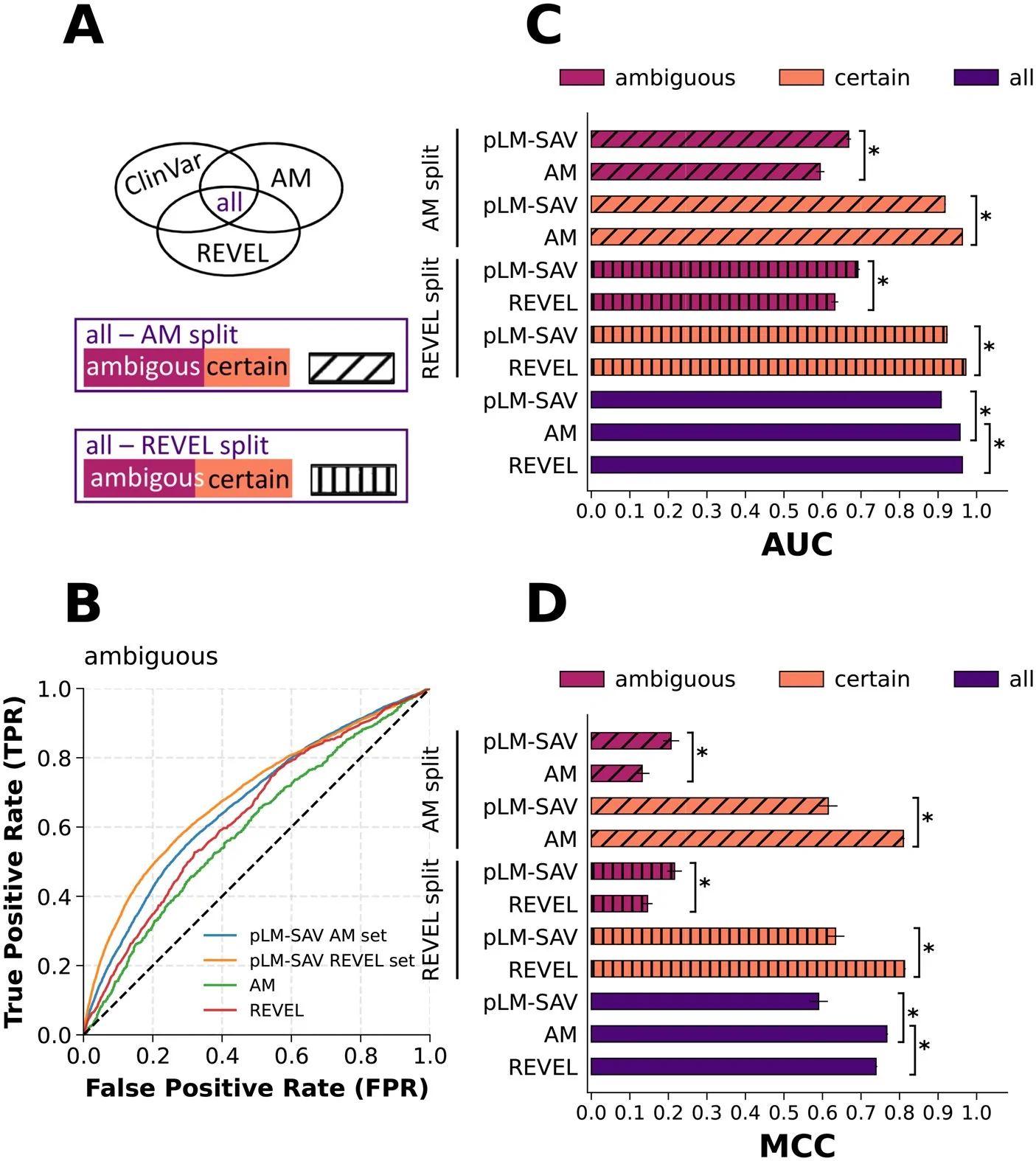
Comparison of the pLM-SAV predictions to AlphaMissense (AM) and REVEL. (A) The test set contained proteins with sequences not similar to those in the training and validation of the pLM-SAV Ankh ‘B’ model, had ClinVar data, AM and REVEL predictions (set CCV-B) denoted as ‘all’. Metrics were calculated for ambiguous, certain and all predictions, splitting the ‘all’ data set according to the ambiguous/certain criteria of AlphaMissense or REVEL. (B) ROC curves for the ambiguous subset, comparing pLM-SAV evaluated on the AM-defined and REVEL-defined ambiguous sets with AM and REVEL, respectively. Additional ROC and TPR curves are shown in Figs S7 and S8. (C) AUC values and (D) MCC values for the ambiguous, certain, and all subsets. Differences compared to the results of the pLM-SAV (Ankh ‘B’) model on the given set were tested according to the method given in Method S1: Performance metrics, exact *P-*value given in Table S9, available as supplementary data at *Bioinformatics Advances* online. Significant difference at *P* < .05 is indicated by *.

The performance of our pLM-SAV predictor was compared to the state-of-the-art SAV predictor AlphaMissense and to the ensemble-based REVEL method. To ensure a fair comparison, independent test sets were selected from our ClinVar set, including only proteins with no sequence similarity to those used in training or validation of pLM-SAV, however, some proteins may have been involved in AlphaMissense or REVEL training (sets CCV-A and CCV-B). These sets contained variants for which both AlphaMissense and REVEL predictions were available (Fig. 4A). Prediction scores (AUC and MCC) across the full datasets were generally lower for pLM-SAV models compared to AlphaMissense and REVEL. When the datasets were divided into ambiguous and certain prediction subsets based on the AM or REVEL predictions, pLM-SAV models consistently outperformed both AlphaMissense and REVEL on the ambiguous subsets across all evaluated metrics. Using pLM-SAV method ‘A’, both Ankh and ProtT5 embeddings yielded higher AUC, MCC, and F1effect scores compared to AlphaMissense on its ambiguous subset (Table S9, available as supplementary data at *Bioinformatics Advances* online). In the case of the ambiguous REVEL subset, only pLM-SAV Ankh exceeded REVEL across all three metrics (AUC, MCC, F1effect), whereas pLM-SAV ProtT5 did not (Table S9, available as supplementary data at *Bioinformatics Advances* online). Similar patterns were observed with method ‘B’, where both pLM-SAV Ankh and ProtT5 outperformed AlphaMissense on its ambiguous variants (Fig. 4C and D). For ambiguous REVEL variants under method ‘B’, pLM-SAV Ankh again outperformed REVEL in all metrics, while ProtT5 showed improvement in AUC only. The ROC and PR curves for the pLM-SAV models and for AM and REVEL can be seen in Fig. 4B and Figs S6 and S8, available as supplementary data at *Bioinformatics Advances* online, which also show also the superiority of the pLM-SAV Ankh models on the ambiguous sets, on the whole sets and the certain subsets AM and REVEL proved to be better.

**Figure 5:**
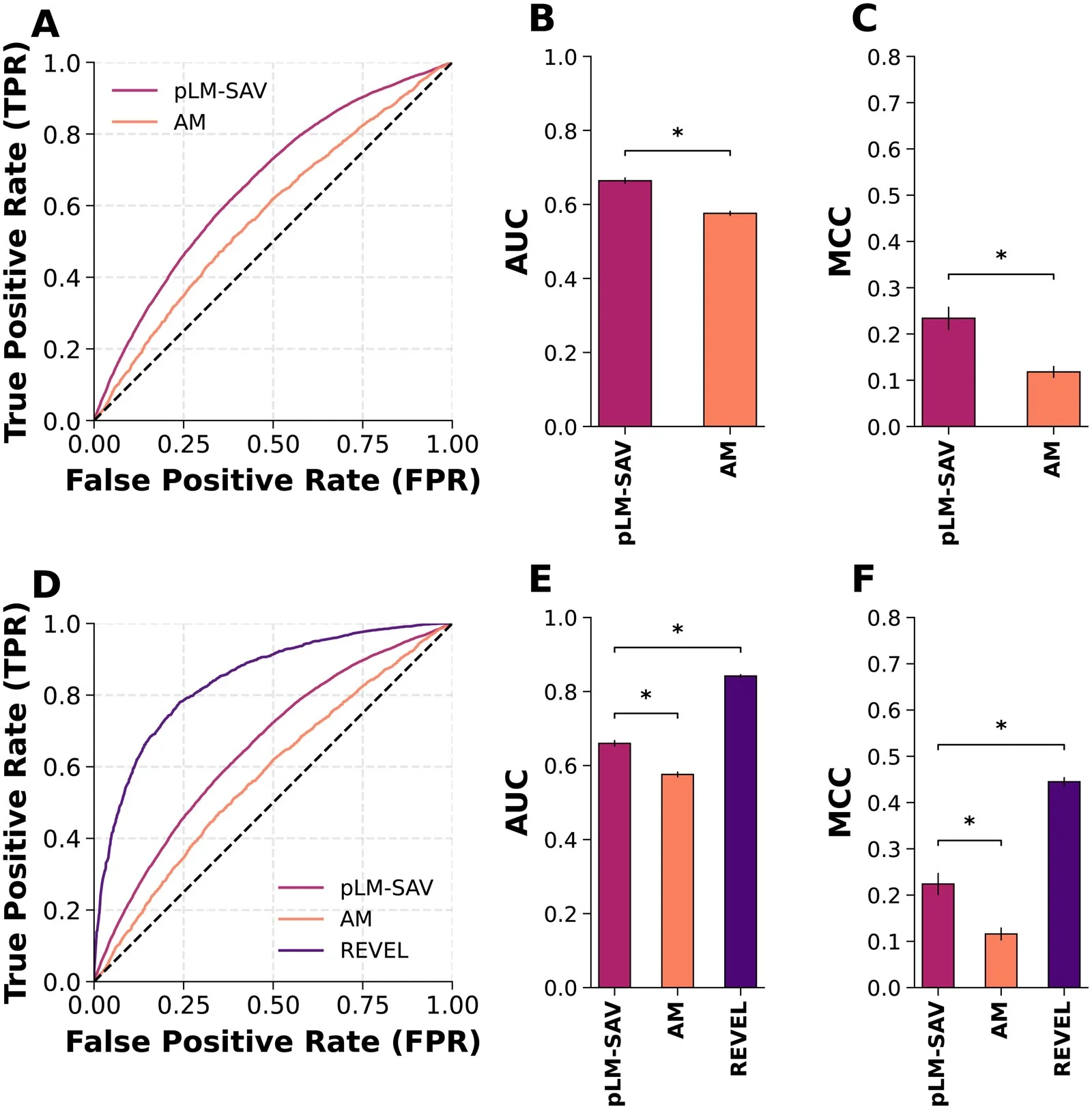
Performance of pLM-SAV, AlphaMissense (AM), and REVEL, where available on the AMambCV and AMambRCV sets. Both datasets comprise variants with ambiguous AM predictions with available ClinVar labels, and AMambRCV set variants have REVEL predictions as well. (A) ROC curves comparing the two methods for the AMambCV set. (B) AMambCV set AUC values, (C) AMambCV set MCC values, (D) AMambRCV set ROC curves for the three methods (E) AMambRCV set AUC values, (F) AMambRCV set MCC values. Differences were tested relative to the pLM-SAV Ankh ‘B’ model as described in Section 2 (Performance metrics); exact *P-*value are reported in Table S10, available as supplementary data at *Bioinformatics Advances* online. Significant differences (*P* < .05) are indicated by an asterisk.

Encouraged by these promising results on the ambiguous subsets of both AlphaMissense and REVEL within the ClinVar test sets, we extended our analysis to larger ambiguous AlphaMissense datasets (AMambCV and AMambRCV). To assess whether pLM-SAV could outperform these methods on a broader scale, we selected the largest possible variant set that included ambiguous AlphaMissense labelled by ClinVar data (AMambCV). pLM-SAV consistently and significantly outperformed AlphaMissense (Fig. 5A–C, Fig. S9A, and Table S10, available as supplementary data at *Bioinformatics Advances* online). Considering its subset, AMambRCV, where REVEL predictions were available as well, similarly pLM-SAV outperformed AM, while REVEL achieved the highest overall performance among the three methods (Fig. 5D–F, Fig. S9B, and Table S10, available as supplementary data at *Bioinformatics Advances* online). The same tendency was observed in case of the largest ambiguous REVEL set with ClinVar labels (RambCV) and its AM prediction-containing subset (RambAMCV) (Fig. S10C–F, Table S10, available as supplementary data at *Bioinformatics Advances* online). Thus, pLM-SAV should not be interpreted as a replacement for established predictors, but rather as a complementary approach that may be most useful when existing methods are missing or provide ambiguous predictions. For variants with ambiguous AlphaMissense scores, REVEL predictions should be considered when available.

### 3.6 Comparison with the ProteinGym v0.1 benchmark

**Figure 6:**
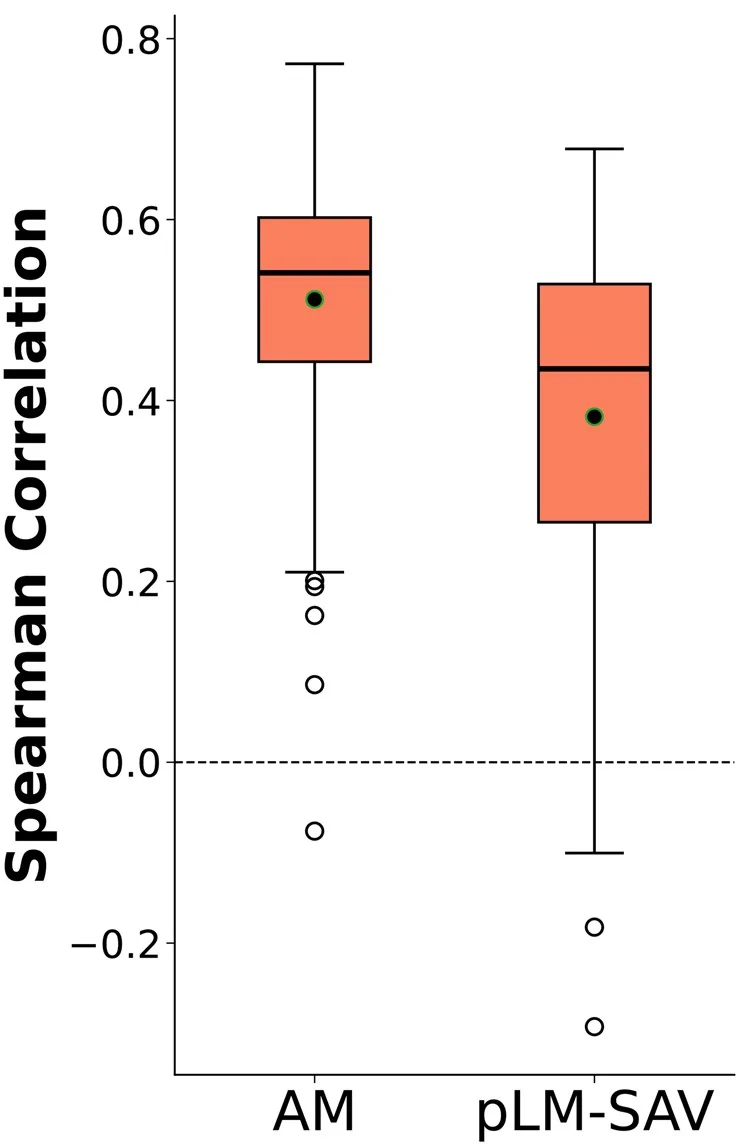
Comparison of the Spearman’s correlation for AlphaMissense and pLM-SAV (method ‘B’ with Ankh). Our pLM-SAV predictions can be found in pLM-SAV_SUPPData.xlsx and on Zenodo.

To evaluate the predictive power of pLM-SAV on a MAVE (or DMS) benchmark, where mutation effects are represented by continuous values rather than binary labels, we used ProteinGym version 0.1 (Notin *et al.* 2022), which was also employed in the AlphaMissense study (see Method S1, available as supplementary data at *Bioinformatics Advances* online). Although the best-performing pLM-SAV model yielded a lower average Spearman rank correlation (0.382) compared to AlphaMissense (0.512) (Fig. 6), pLM-SAV performed in a similar range as the unsupervised ESM-1b on this benchmark (0.358) (Rives *et al.* 2021).

## 4 Discussion

In this study, we demonstrated that a simple Δ-embedding-based architecture, pLM-SAV, can achieve performance comparable to more complex SAV effect prediction methods. By leveraging the representational power of pLMs, and focusing on the difference between wild-type and mutant embeddings, we showed that even a lightweight convolutional neural network can extract informative features for binary classification of SAVs.

Our results confirm the viability of using Δ-embeddings for SAV effect prediction. The best-performing pLM-SAV models (particularly those using the Ankh language model and trained with method ‘B’) consistently achieved strong predictive performance on the Eff10k test folds and on independent ClinVar subsets (Fig. 2). Notably, pLM-SAV showed solid performance across both human and non-human proteins in the PMD4k benchmark and performed in a similar range to VESPA and SNAP2 (Fig. 3). This suggests that Δ-embeddings alone can efficiently capture the functional effects of mutations, even without explicit structural, evolutionary, or annotation-based features. This distinguishes pLM-SAV from approaches like VESPA, which combine pLM embeddings with substitution matrices and manually curated features such as BLOSUM scores and amino acid probabilities. In contrast, pLM-SAV relies solely on Δ-embeddings, enabling a leaner and more interpretable architecture, while also achieving comparable or shorter run times (Table S11, available as supplementary data at *Bioinformatics Advances* online). It is important to note that while we highlight the strong performance of pLM-SAV with its lightweight setup, developing and training PLMs is a complex and computationally expensive task. These models encode information with a level of complexity potentially comparable to that extracted from MSAs and other biological data sources.

To further support that Δ-embeddings capture mutation-induced changes in pLM embedding space, we performed additional quantitative analyses of Δ-embedding magnitudes. Across 99 926 Eff10k variants, the average absolute Δ-embedding signal was strongest at the mutated residue, but neighbouring residues also contributed, supporting the use of a local 7-residue window (Fig. S2, available as supplementary data at *Bioinformatics Advances* online). Consistent with this, aggregation of absolute Δ-embedding values at the per-dimension and per-variant levels confirmed measurable mutation-induced embedding shifts for both Ankh and ProtT5 (Table S5, available as supplementary data at *Bioinformatics Advances* online). In addition, effect variants showed larger summed squared Δ-embedding differences than neutral variants, and prediction errors were associated with the magnitude of these differences: false negatives tended to have smaller Δ-embedding shifts, whereas false positives tended to have larger shifts (Fig. S3, available as supplementary data at *Bioinformatics Advances* online). These analyses indicate that single amino acid substitutions induce detectable local changes in pLM embedding space and that these changes contain predictive information for variant effect classification, although their magnitude alone does not provide a specific or fully discriminative signal. We also emphasize that the Δ-embedding-magnitude analyses are descriptive rather than mechanistic. Although effect variants tend, on average, to show larger local embedding shifts, the substantial overlap with neutral variants means that Δ-embedding magnitude alone is neither a specific biological readout of mutation effect nor a sufficient classifier. Neutral variants with large shifts may contribute to false-positive predictions, whereas effect variants with small shifts may contribute to false-negative predictions. pLM-SAV uses the full signed and multidimensional local Δ-embedding pattern rather than an aggregated magnitude, but this does not remove the limitation of interpreting Δ-embeddings as a uniquely specific representation of biological effect.

Although pLM-SAV showed lower overall performance than AlphaMissense (Cheng *et al.* 2023) and REVEL (Ioannidis *et al.* 2016) on the complete ClinVar-derived benchmark datasets (Fig. 4), its performance on ambiguity-defined subsets varied depending on which predictor was considered ambiguous and which comparison methods were available for the same variants (Figs 4 and 5, Figs S9 and S10, available as supplementary data at *Bioinformatics Advances* online). On the larger ClinVar-labelled AlphaMissense-ambiguous set, pLM-SAV outperformed AlphaMissense. However, within the subset for which REVEL predictions were available, REVEL achieved the highest overall performance although pLM-SAV outperformed AM as well. Since REVEL outperformed both pLM-SAV and AlphaMissense on this subset, we incorporated REVEL predictions into our web application (alphamissense.hegelab.org) to enhance its utility (Tordai *et al.* 2024). Conversely, pLM-SAV outperformed REVEL on the broad REVEL-ambiguous ClinVar set, whereas AlphaMissense performed best in the subset of REVEL-ambiguous variants for which AlphaMissense predictions were available (Fig. S10C–F, Table S10, available as supplementary data at *Bioinformatics Advances* online), while pLM-SAV outperformed REVEL. On the jointly AlphaMissense- and REVEL-ambiguous subset, which contains variants where both of these predictors struggle, all the predictions were much more similar and the difference in some cases was not significant (Fig. S10A and B, Table S10, available as supplementary data at *Bioinformatics Advances* online); neither result supports a general performance advantage of pLM-SAV.

These analyses define a more limited and practical role for pLM-SAV. It can provide an additional prediction in selected ambiguity-defined settings, particularly when a leading predictor is ambiguous and a stronger alternative prediction is unavailable. However, pLM-SAV should not be considered a replacement for AlphaMissense or REVEL, and its use should be interpreted together with available predictions from these methods. The distinct behaviour of pLM-SAV across these subsets is consistent with a partially complementary prediction signal but does not establish that Δ-embeddings are intrinsically superior to alternative representations. In these AM and/or REVEL ambiguous subsets the distribution of the pLM-SAV method ‘B’ with Ankh predictions were calculated and is shown in Fig. S11, available as supplementary data at *Bioinformatics Advances* online for the AMambCV, RambCV and AMambRambCV sets. In all three subsets, prediction frequencies are highest at values farther from the 0.41–0.66 interval spanned by the standard classification thresholds of the nine pLM-SAV method ‘B’ Ankh models, rather than around this interval, indicating that these variants are generally not close to the pLM-SAV decision boundary. This may help explain the better performance of pLM-SAV in some of these subsets.

It is important to emphasize that our approach is trained on labelled data derived from the Eff10k dataset (Hecht *et al.* 2015), where class labels indicate whether a mutation has any measurable effect, regardless of its pathogenicity. In contrast, the ClinVar-based evaluation relies on a strict benign versus pathogenic distinction. Thus, the Eff10k and ProteinGym analyses primarily assess general functional or fitness-related variant effects, whereas the ClinVar analyses assess clinical pathogenicity. These endpoints are related but not interchangeable, and differences between them may contribute to the lower performance observed on some ClinVar-based benchmarks. This mismatch in classification categories could partly explain the lower performance observed in some ClinVar test sets, especially in the CV-A subset, which is significantly smaller and more label-imbalanced than CV-B. Lastly, our model’s performance on the ProteinGym benchmark further supports the relevance of Δ-embeddings. Although pLM-SAV did not reach the performance of AlphaMissense in this benchmark, it matched the unsupervised ESM-1b model (Rives *et al.* 2021), suggesting that it captures biologically meaningful variation despite being trained on binary labels. These observations highlight that current benchmarks may impose a performance ceiling due to dataset limitations such as label ambiguity, imbalance, and inconsistent annotation standards. However, we believe that further improvements remain possible through better handling of ambiguous variants and the integration of complementary features such as structure-aware representations.

While pLM-SAV was developed for SAVs, the approach could be extended to more complex variants such as insertions, deletions, and fusions. In these cases, embedding pairs cannot be subtracted within the altered region, but Δ-embeddings from surrounding residues could still be computed and aggregated for classification. Given the contextual encoding of pLMs, effects of such variants are likely to manifest outside the mutated region as well. However, using larger windows may introduce noise, and the lack of high-quality training data currently limits practical implementation.

Interestingly, the Δ-embedding signal often extends beyond the mutated residue (Fig. 1 and Fig. S2, available as supplementary data at *Bioinformatics Advances* online), suggesting that pLM-based representations capture contextual dependencies. This raises the possibility that predictions may be modulated by other sequence variations–benign or otherwise–appearing elsewhere in the protein. However, the usually low Δ-embedding values at positions distant from the mutation site (Fig. 1), together with our preliminary results (Table S12, available as supplementary data at *Bioinformatics Advances* online), suggest that such distant residues do not systematically influence the model’s output.

Overall, pLM-SAV provides a compact Δ-embedding-based approach for SAV-effect prediction that requires no handcrafted structural, evolutionary, or annotation-derived input features. Its classifier is lightweight once embeddings are available, and their generation from the sequence is straightforward, although generating wild-type and mutant pLM embeddings remains the main computational cost. The results support Δ-embeddings as a useful mutation-centred representation, while also showing that their magnitude alone is not biologically specific and that their value depends on the dataset and comparison setting. Future work may explore how Δ-embedding features can complement other sources of information in variant-effect prediction.

## Supplementary Material

vbag195_Supplementary_Data

## Data Availability

The data and code underlying this article are available in Zenodo at https://zenodo.org/, and can be accessed with https://doi.org/10.5281/zenodo.15502498. The associated web server is freely available at https://alphamissense.hegelab.org.

## References

[vbag195-B1] Adzhubei IA , Schmidt S, Peshkin L et al A method and server for predicting damaging missense mutations. Nat Methods 2010;7:248–9. 10.1038/nmeth0410-248 20354512 PMC2855889

[vbag195-B2] Arnold S , Buchanan DD, Barker M et al Classifying MLH1 and MSH2 variants using bioinformatic prediction, splicing assays, segregation, and tumor characteristics. Hum Mutat 2009;30:757–70. 10.1002/humu.20936 19267393 PMC2707453

[vbag195-B3] Bairoch A , Apweiler R. The SWISS-PROT protein sequence database and its supplement TrEMBL in 2000. Nucleic Acids Res 2000;28:45–8. 10.1093/nar/28.1.45 10592178 PMC102476

[vbag195-B4] Brandes N , Goldman G, Wang CH et al Genome-wide prediction of disease variant effects with a deep protein language model. Nat Genet 2023;55:1512–22. 10.1038/s41588-023-01465-0 37563329 PMC10484790

[vbag195-B5] Capriotti E , Calabrese R, Casadio R. Predicting the insurgence of human genetic diseases associated to single point protein mutations with support vector machines and evolutionary information. Bioinformatics 2006;22:2729–34. 10.1093/bioinformatics/btl423 16895930

[vbag195-B6] Cheng J , Novati G, Pan J et al Accurate proteome-wide missense variant effect prediction with AlphaMissense. Science (1979) 2023;381:eadg7492. 1126/SCIENCE.ADG7492 10.1126/science.adg7492 37733863

[vbag195-B7] Dimmer EC , Huntley RP, Alam-Faruque Y et al The UniProt-GO annotation database in 2011. Nucleic Acids Res 2012;40:D565–70. 10.1093/NAR/GKR1048 22123736 PMC3245010

[vbag195-B8] Drozda JP , Roach J, Forsyth T et al Constructing the informatics and information technology foundations of a medical device evaluation system: a report from the FDA unique device identifier demonstration. J Am Med Inform Assoc 2018;25:111–20. 10.1093/jamia/ocx041 28472359 PMC7647129

[vbag195-B9] Elnaggar A , Essam H, Salah-Eldin W et al Ankh ☥: optimized protein language model unlocks General-Purpose modelling. bioRxiv, 2023, 2023.01.16.524265, preprint: not peer reviewed.

[vbag195-B10] Elnaggar A , Heinzinger M, Dallago C et al ProtTrans: toward understanding the language of life through self-supervised learning. IEEE Trans Pattern Anal Mach Intell 2022;44:7112–27. 10.1109/TPAMI.2021.3095381 34232869

[vbag195-B11] Findlay GM , Daza RM, Martin B et al Accurate classification of BRCA1 variants with saturation genome editing. Nature 2018;562:217–22. 10.1038/s41586-018-0461-z 30209399 PMC6181777

[vbag195-B12] Gong J , Jiang L, Chen Y et al THPLM: a sequence-based deep learning framework for protein stability changes prediction upon point variations using pretrained protein language model. Bioinformatics 2023;39:btad646. 10.1093/bioinformatics/btad646 PMC10627365 37874953

[vbag195-B13] Hamosh A , Scott AF, Amberger JS et al Online mendelian inheritance in man (OMIM), a knowledgebase of human genes and genetic disorders. Nucleic Acids Res 2005;33:D514–17. 10.1093/NAR/GKI033 15608251 PMC539987

[vbag195-B14] Hecht M , Bromberg Y, Rost B. Better prediction of functional effects for sequence variants. BMC Genomics 2015;16 (Suppl. 8):S1. 10.1186/1471-2164-16-S8-S1 PMC4480835 26110438

[vbag195-B15] Heinzinger M , Rost B. Teaching AI to speak protein. Curr Opin Struct Biol 2025;91:102986. 10.1016/J.SBI.2025.102986 39985945

[vbag195-B16] Ioannidis NM , Rothstein JH, Pejaver V et al REVEL: an ensemble method for predicting the pathogenicity of rare missense variants. Am J Hum Genet 2016;99:877–85. 10.1016/j.ajhg.2016.08.016 27666373 PMC5065685

[vbag195-B17] Karczewski KJ , Francioli LC, Tiao G et al; Genome Aggregation Database Consortium. The mutational constraint spectrum quantified from variation in 141,456 humans. Nature 2020;581:434–43. 10.1038/s41586-020-2308-7 32461654 PMC7334197

[vbag195-B18] Kawabata T , Ota M, Nishikawa K. The protein mutant database. Nucleic Acids Res 1999;27:355–7. 10.1093/nar/27.1.355 9847227 PMC148182

[vbag195-B19] Lek M , Karczewski KJ, Minikel EV et al; Exome Aggregation Consortium. Analysis of protein-coding genetic variation in 60,706 humans. Nature 2016;536:285–91. 27535533 10.1038/nature19057 PMC5018207

[vbag195-B20] Lin W , Wells J, Wang Z et al Enhancing missense variant pathogenicity prediction with protein language models using VariPred. Sci Rep 2024;14:8136. 10.1038/s41598-024-51489-7 38584172 PMC10999449

[vbag195-B21] Marquet C , Heinzinger M, Olenyi T et al Embeddings from protein language models predict conservation and variant effects. Hum Genet 2022;141:1629–47. 10.1007/s00439-021-02411-y 34967936 PMC8716573

[vbag195-B22] Ng PC , Henikoff S. SIFT: predicting amino acid changes that affect protein function. Nucleic Acids Res 2003;31:3812–4. 10.1093/nar/gkg509 12824425 PMC168916

[vbag195-B23] Notin P , Dias M, Frazer J et al Tranception: protein fitness prediction with autoregressive transformers and inference-time retrieval. In: Chaudhuri K, Jegelka S, Song L (eds.), Proceedings of the 39th International Conference on Machine Learning, Vol. 162, Baltimore, MD: PMLR, 2022, 16990–7017. 10.48550/arXiv.2205.13760

[vbag195-B24] Paszke A , Gross S, Massa F et al PyTorch: an imperative style, High-Performance deep learning library. In: Wallach H, Larochelle H, Beygelzimer A (eds.), Advances in Neural Information Processing Systems. Vol 32. Red Hook, NY: Curran Associates, Inc., 2019.

[vbag195-B25] Radjasandirane R , Cretin G, Diharce J et al PATHOS: predicting variant pathogenicity by combining protein language models and biological features. Artif Intell Life Sci 2026;9:100165. 10.1016/j.ailsci.2026.100165

[vbag195-B26] Rives A , Meier J, Sercu T et al Biological structure and function emerge from scaling unsupervised learning to 250 million protein sequences. Proc Natl Acad Sci U S A 2021;118:e2016239118. 10.1073/pnas.2016239118 PMC8053943 33876751

[vbag195-B27] Sattar S , Kenis C, Haase K et al Falls in older patients with cancer: Nursing and Allied Health Group of International Society of Geriatric Oncology review paper. J Geriatr Oncol 2020;11:1–7. 10.1016/j.jgo.2019.03.020 30956135

[vbag195-B28] Savojardo C , Manfredi M, Martelli PL et al DDGemb: predicting protein stability change upon single- and multi-point variations with embeddings and deep learning. Bioinformatics 2024;41:btaf019. 10.1093/bioinformatics/btaf019 PMC11783275 39799516

[vbag195-B29] Tordai H , Torres O, Csepi M et al Analysis of AlphaMissense data in different protein groups and structural context. Sci Data 2024;11:495. 10.1038/S41597-024-03327-8 38744964 PMC11094042

[vbag195-B30] Wang D , Pourmirzaei M, Abbas UL et al S-PLM: structure-aware protein language model via contrastive learning between sequence and structure. Adv Sci (Weinh) 2025;12:e2404212. 10.1002/ADVS.202404212 39665266 PMC11791933

[vbag195-B31] Yan Z , Ge F, Liu Y et al TransEFVP: a two-stage approach for the prediction of human pathogenic variants based on protein sequence embedding fusion. J Chem Inf Model 2024;64:1407–18. 10.1021/acs.jcim.3c02019 38334115

