# Supplementary material for: pLM-SAV: a Δ-embedding approach for predicting pathogenic single amino acid variants
Source: Bioinform Adv. 2026 Jul 15;6(1):vbag195. doi: 10.1093/bioadv/vbag195 (PMC13581460; doi:10.1093/bioadv/vbag195)
Supplement: Supplementary file 1 [file vbag195_supplementary_data.zip › pLM-SAV-SUPPL-20260623-final.pdf]

SUPPLEMENTARY MATERIAL

pLM-SAV: A Δ-Embedding Approach for Predicting Pathogenic Single Amino Acid Variants

Orsolya Gereben, Hedvig Tordai, Lana Khamisi, Erda Qorri, Tamás Hegedűs

Method S1. The corrected sample standard deviation of performance was determined from the results of the best models, based on 10 models for method ‘A’ and 9 models for method ‘B’ in the case of pLM-SAV, and bootstrapping (n=1,000) was used in case of AlphaMissense and REVEL data.

To assess whether the difference between results were significant, first the normality of the distributions were tested with the Anderson-Darling test at significance level 15 %. If both distributions proved to be normal, then Student’s *t*-test otherwise Wilcoxon rank sum test was performed. In both cases the two samples considered to be different if the *p*-value was below 0.05.

For the ProteinGym v0.1 benchmark, performance was evaluated using the methodology described in the original study (Notin *et al.* 2022) and AlphaMissense [(Cheng et al. 2023)]. Since this benchmark includes variants with multiple amino acid mutations, raw scores were calculated separately for each mutation within a multi-mutant variant (assuming independent effects, although this assumption may not always hold). These scores were summed to obtain a combined score. A sigmoid function was applied to the summed score, generating a single prediction per multi-mutant variant.

Spearman's rank correlation was computed separately for each assay with the best models of method ‘B’ Ankh. The data is given in file pLM-SAV_SUPPData.xlsx. The results were then averaged at the protein level, as some proteins had multiple assays. A final mean of Spearman correlation was calculated across 72 proteins in the benchmark. To ensure that a positive correlation reflects agreement between assay values and predictions, the sign of the Spearman correlation coefficients was inverted. Although some studies (including AlphaMissense) report the absolute value of Spearman correlation instead of the signed value, we followed the ProteinGym v0.1 benchmark recommendations and retained the original signed correlation. Consequently, the AlphaMissense Spearman correlation for this benchmark was recalculated for consistency with this methodology.

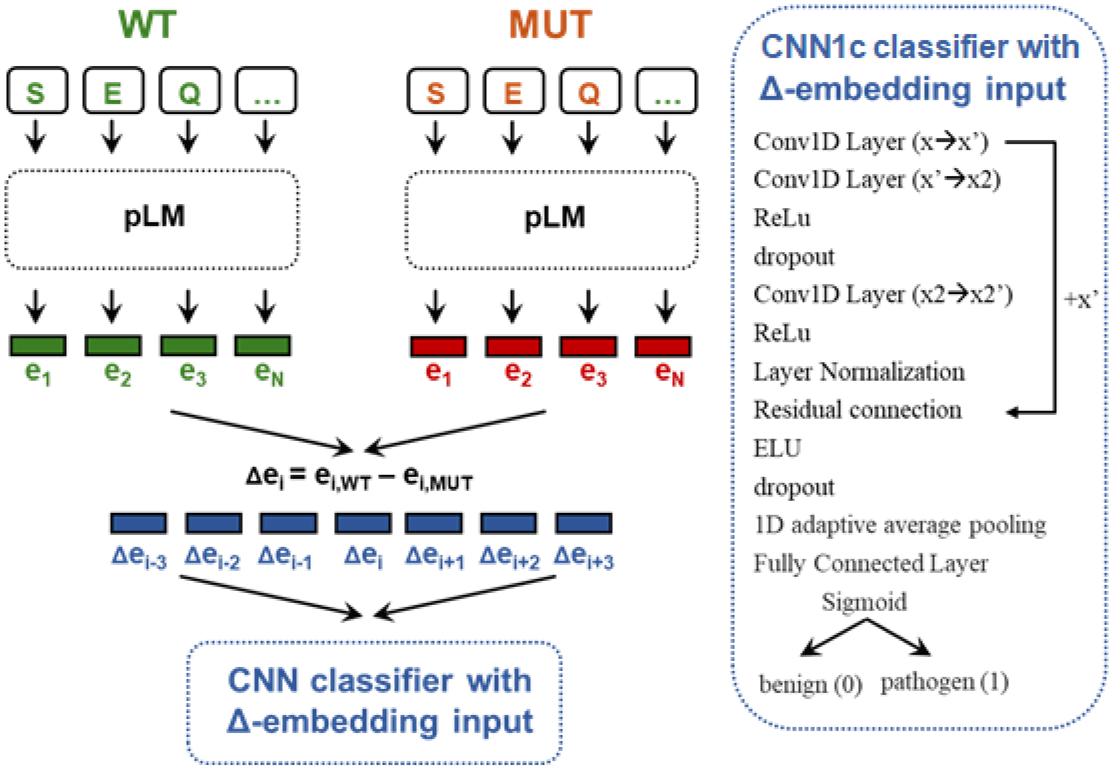

Figure S1. Schematic representation of pLM-SAV architecture. WT: wild type, MUT: mutant, pLM: protein language model, e_i: embedding of a residue, Δe: delta embedding calculated as $embedding_{i,WT}$ - $embedding_{i,MUT}$, CNN: convolutional neural network, ReLu: rectified linear unit, ELU: exponential linear unit. The most successful CNN1c architecture consists of two 1D convolutional layers, each followed by a ReLU (Rectified Linear Unit) activation function, a widely used non-linearity in neural networks that outputs zero for negative values and the identity for positive values (Paszke *et al.* 2019), scaling the output to 8 channels with a kernel size of three. The convolution is performed in the window length dimension. After the second convolution, layer normalization is applied, followed by a residual connection, adding input to the output of a layer, to improve gradient flow, learning, and performance. The ELU (Exponential Linear Unit) activation function (Paszke *et al.* 2019) is then used, which can produce negative outputs and helps improve learning dynamics compared to ReLU. To further reduce dimensionality, global pooling is applied – this operation condenses each feature map to a single value – before passing the output through a fully connected layer with sigmoid activation. Additionally, two dropout layers are incorporated to mitigate overfitting by randomly deactivating neurons during training. The neural network was implemented using PyTorch (Paszke *et al.* 2019), and the class definitions are available on Zenodo (https://doi.org/10.5281/zenodo.15502498), in the *mynn.py* file.

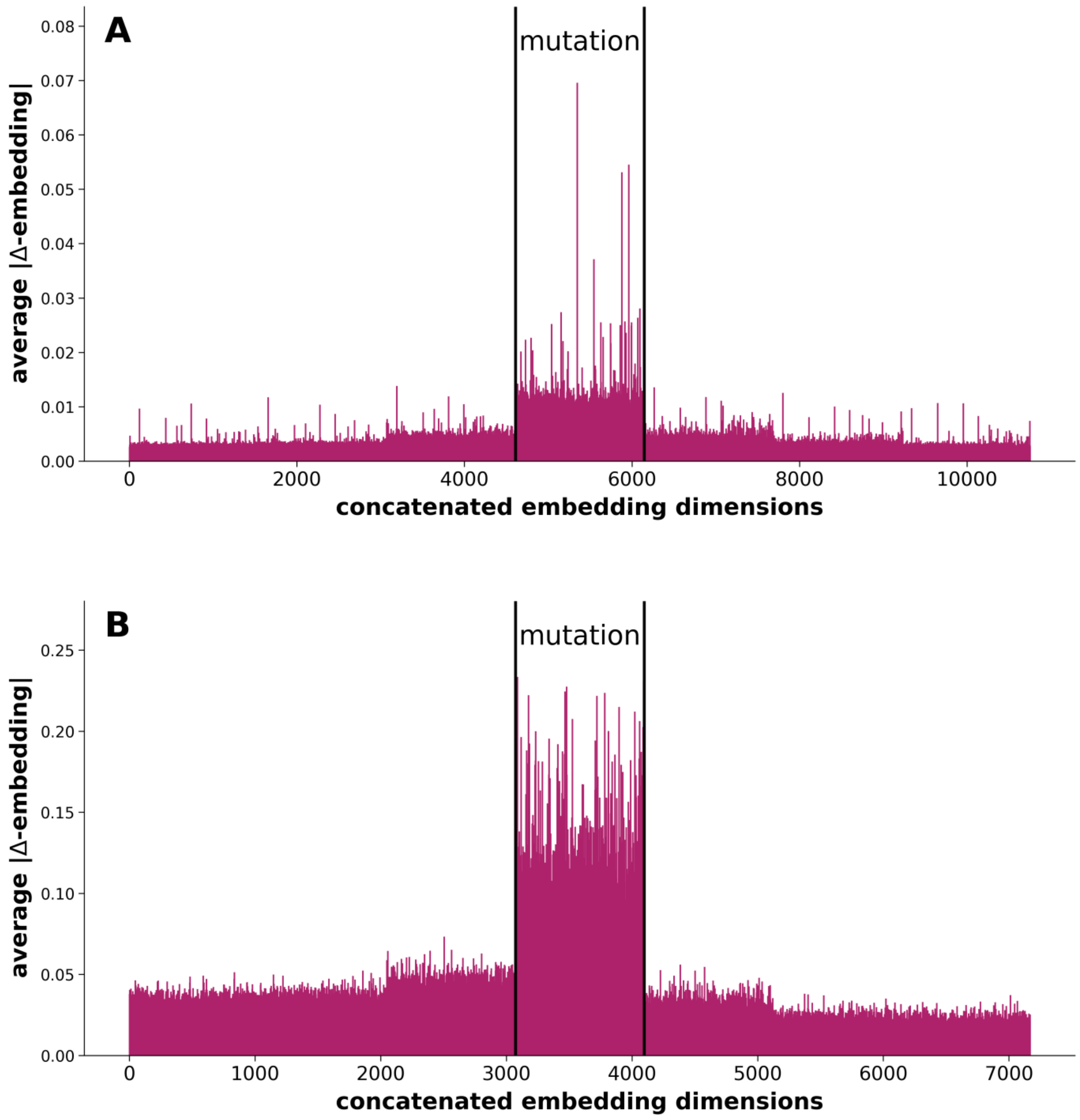

Figure S2. Average absolute Δ-embedding values across the concatenated embedding dimensions of the 7 amino acid window centered on the mutation. The analysis was performed on N = 99,926 variants from the Eff10k dataset. For this analysis, the computed Δ-embedding vectors had dimensions of (N, 7×D), where 7xD=7×1,536=10,752 for Ankh and 7×1,024=7,168 for ProtT5, using absolute values. Averaging across variants yielded a vector of length 7×D, which is visualized here for Ankh (**A**) and ProtT5 (**B**) language models. The largest contributions are observed at the central position corresponding to the mutation site, although neighboring residues also contribute. ProtT5 exhibits generally larger absolute Δ-embedding values than Ankh. However, despite these larger mutation-induced embedding shifts, Ankh provides superior performance in SAV prediction.

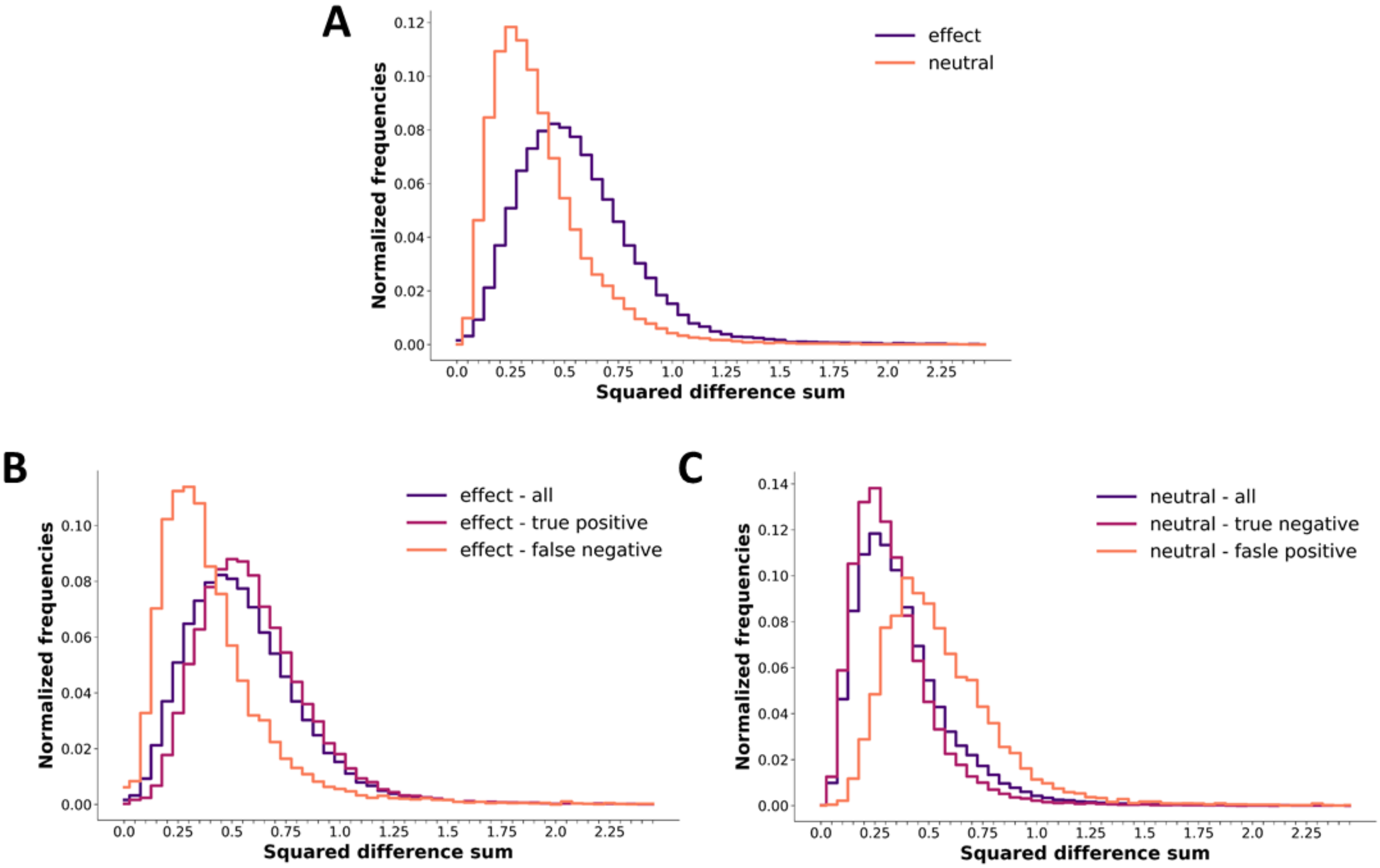

Figure S3. Analysis of the summed squared differences within the 7-amino acid Δ-embedding window for the Eff10k data set. **(A)** Data set split according to effect and neutral variants indicate larger difference between the wild type and mutant embeddings for the effect variants than for the neutral variants illustrated by the normalized histograms. **(B)** Effect variants divided into true positive (larger subset) and false negative (smaller subset) predictions by the Ankh 'A' model reveal that true positives are enriched at the higher end of the distribution, whereas false negatives are shifted toward lower values (i.e., smaller embedding differences). **(C)** Neutral variants divided into true negative (larger subset) and false positive (smaller subset) predictions show the opposite trend, with true negatives enriched at lower values and false positives shifted toward higher values (i.e., larger embedding differences).

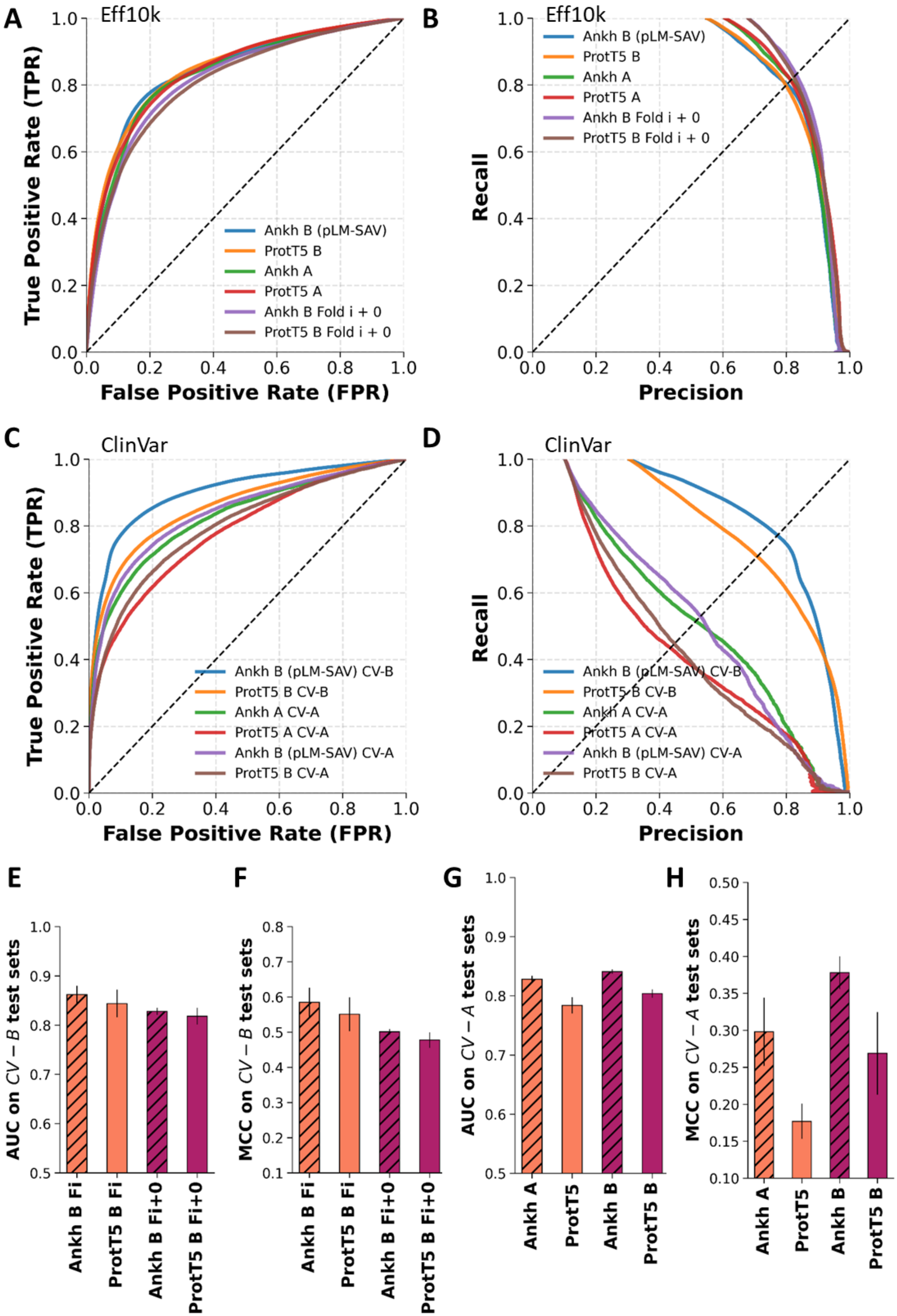
A
Eff10k
True Positive Rate (TPR)
False Positive Rate (FPR)
Ankh B (pLM-SAV)
ProtT5 B
Ankh A
ProtT5 A
Ankh B Fold i + 0
ProtT5 B Fold i + 0
B
Eff10k
Recall
Precision
Ankh B (pLM-SAV)
ProtT5 B
Ankh A
ProtT5 A
Ankh B Fold i + 0
ProtT5 B Fold i + 0
C
ClinVar
True Positive Rate (TPR)
False Positive Rate (FPR)
Ankh B (pLM-SAV) CV-B
ProtT5 B CV-B
Ankh A CV-A
ProtT5 A CV-A
Ankh B (pLM-SAV) CV-A
ProtT5 B CV-A
D
ClinVar
Recall
Precision
Ankh B (pLM-SAV) CV-B
ProtT5 B CV-B
Ankh A CV-A
ProtT5 A CV-A
Ankh B (pLM-SAV) CV-A
ProtT5 B CV-A
E
AUC on CV − B test sets
Ankh B Fi
ProtT5 B Fi
Ankh B Fi+0
ProtT5 B Fi+0
F
MCC on CV − B test sets
Ankh B Fi
ProtT5 B Fi
Ankh B Fi+0
ProtT5 B Fi+0
G
AUC on CV − A test sets
Ankh A
ProtT5
Ankh B
ProtT5 B
H
MCC on CV − A test sets
Ankh A
ProtT5
Ankh B
ProtT5 B

Figure S4. Receiver operating characteristic (ROC) and precision-recall (PR) curves for the Eff10k and ClinVar test sets shown in Figure 2 and Table S6. (**A**) ROC curves for the concatenated Eff10k test sets. (**B**) PR curves for the concatenated Eff10k test sets. (**C**) ROC curves for the ClinVar test sets. (**D**) PR curves for the ClinVar test sets. In all cases, method B using the Ankh language model shows the best performance. **(E, F)** AUC and MCC values indicate that adding the large Fold 0 set to Fold i as a combined test set (Fi+0) reduces predictive performance, suggesting that Fold 0 represents a more challenging subset. **(G, H)** To directly compare methods 'A' and 'B', method 'B' was also evaluated on the CV-A test set. Under these matched conditions, method 'B' achieved higher performance than method 'A', confirming the advantage of method 'B'.

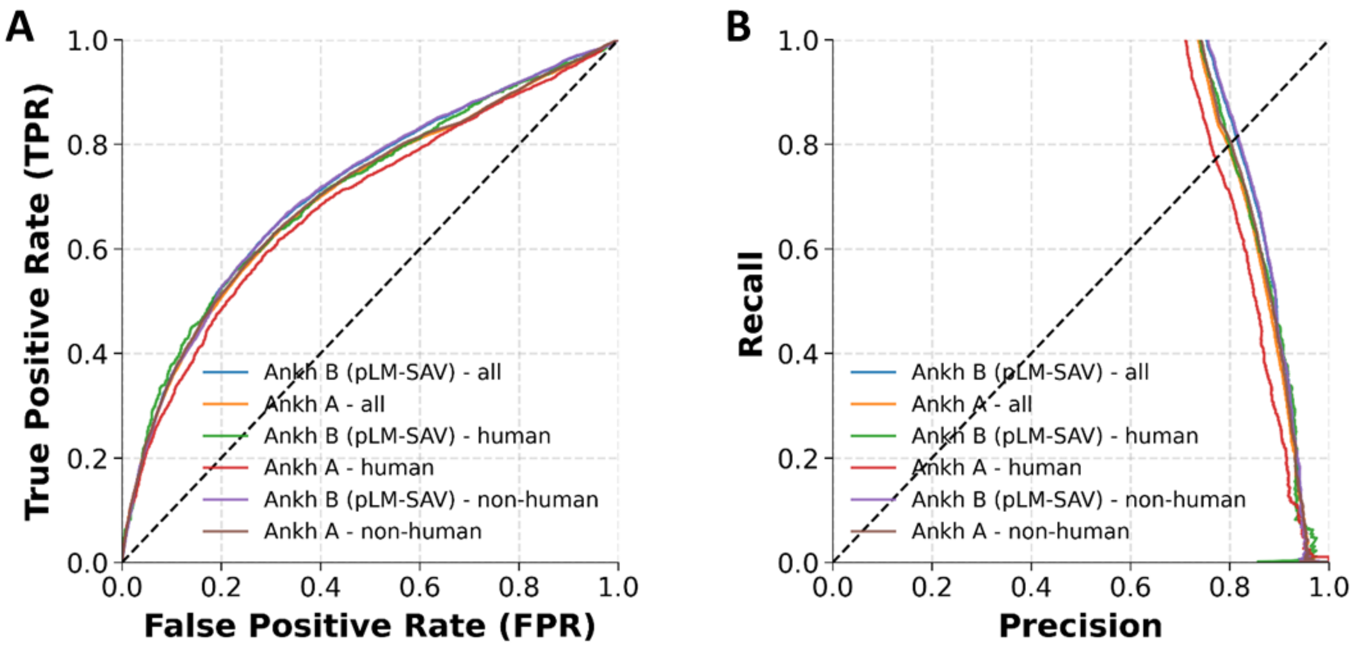

Figure S5. Receiver operating characteristic (ROC) and precision–recall (PR) curves for pLM-SAV predictions on the PMD4k dataset, including the full set and the human/non-human split, as shown in Figure 3 and Tables S7-S8. (**A**) ROC curves for the concatenated PMD4k dataset and its human and non-human subsets and (**B**) corresponding PR curves. In all cases, method B using the Ankh language model shows the best performance.

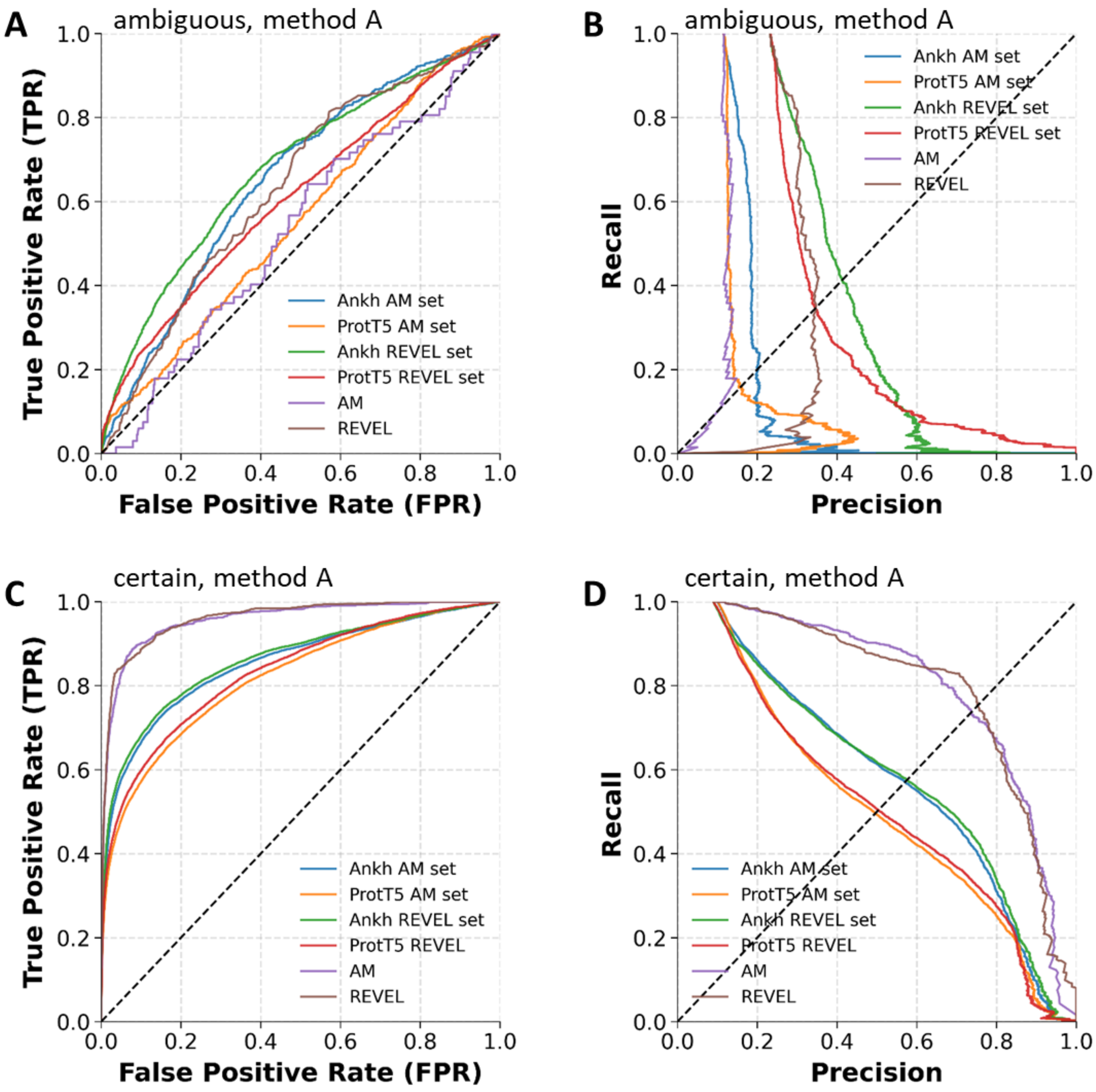

Figure S6. Receiver operating characteristic (ROC) and precision–recall (PR) curves for pLM-SAV method A, AlphaMissense, and REVEL on the CCV-A dataset, stratified into ambiguous and certain subsets based on AlphaMissense or REVEL classifications, as also reported in Table S9. (**A**) ROC curves for the ambiguous subsets; (**B**) PR curves for the ambiguous subsets; (**C**) ROC curves for the certain subsets; (**D**) PR curves for the certain subsets. The Ankh language model consistently outperforms ProtT5. On the ambiguous subsets, our method A outperforms both AlphaMissense and REVEL, whereas on the certain subsets, AlphaMissense and REVEL achieve higher performance than method A.

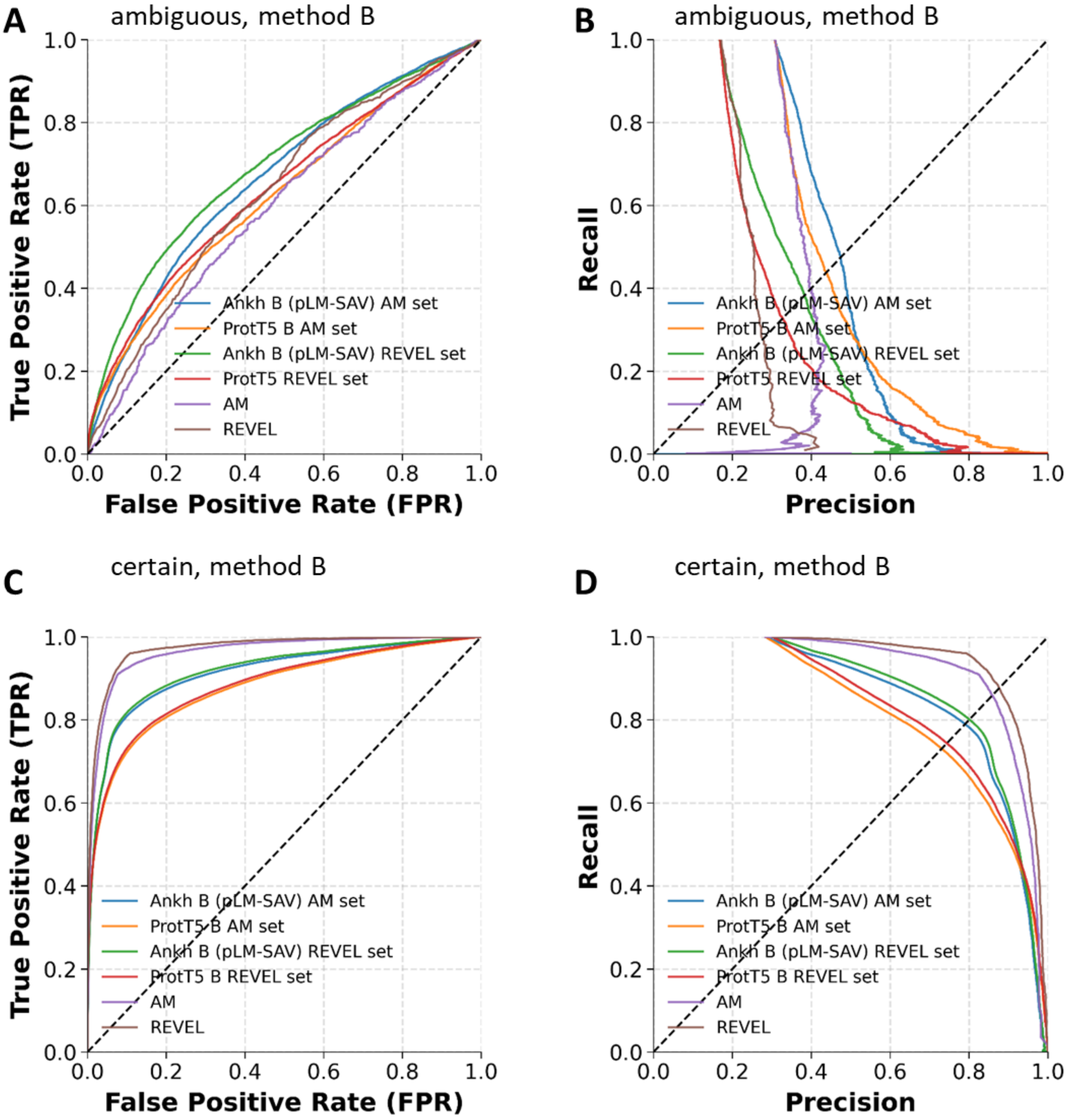

Figure S7. Receiver operating characteristic (ROC) and precision–recall (PR) curves for pLM-SAV method B, AlphaMissense, and REVEL on the CCV-B dataset, stratified into ambiguous and certain subsets based on AlphaMissense or REVEL classifications, as shown in Figure 4 and Table S9. (**A**) ROC curves for the ambiguous subsets; (**B**) PR curves for the ambiguous subsets; (**C**) ROC curves for the certain subsets; (**D**) PR curves for the certain subsets. The Ankh language model consistently outperforms ProtT5. On the ambiguous subsets, pLM-SAV B outperforms both AlphaMissense and REVEL, whereas on the certain subsets, AlphaMissense and REVEL achieve higher performance than pLM-SAV.

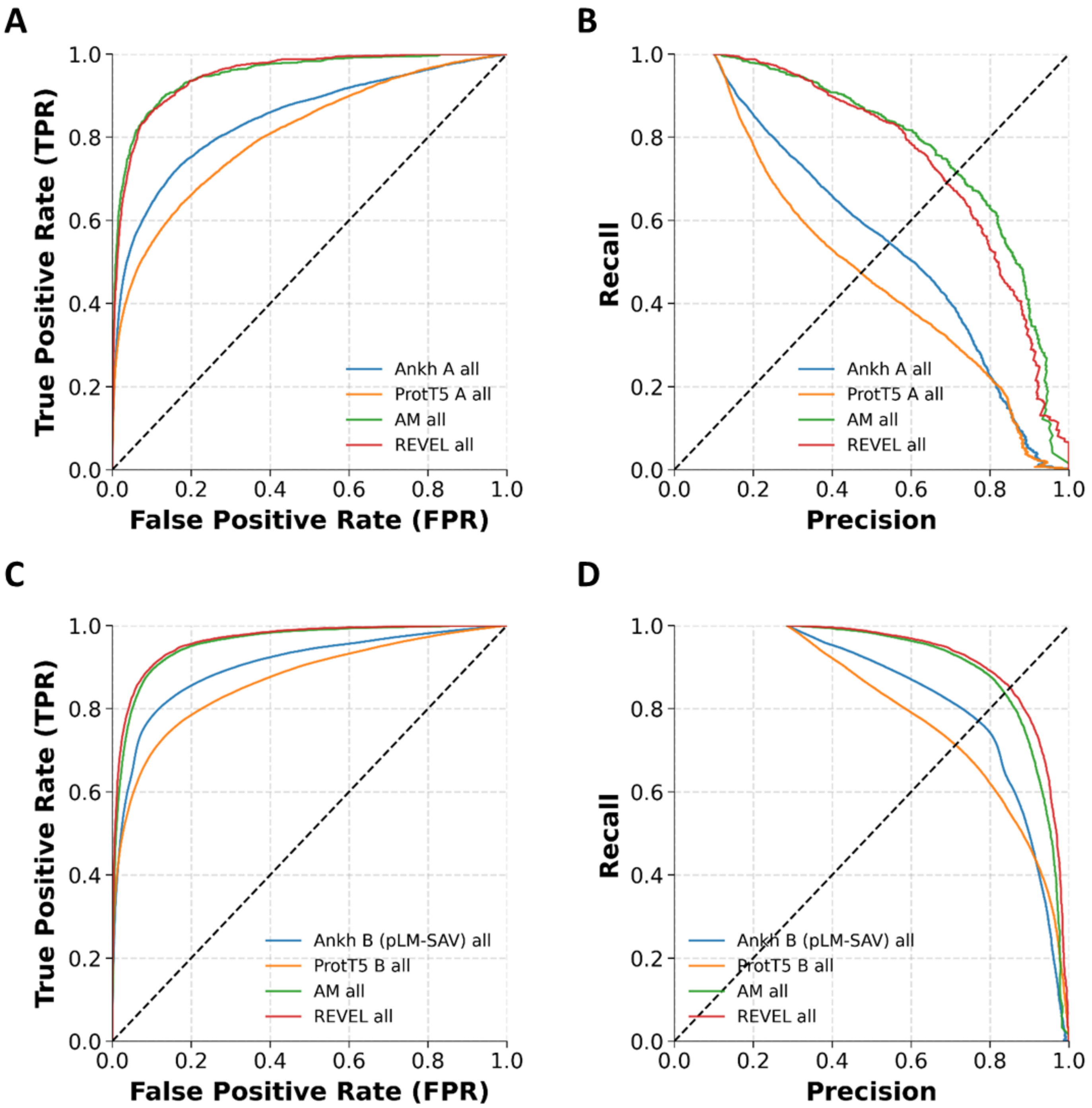

Figure S8. Receiver operating characteristic (ROC) and precision–recall (PR) curves for pLM-SAV methods 'A' and 'B', together with AlphaMissense and REVEL, on the CCV-A and CCV-B datasets, as shown in Figure 4 and Table S9. (**A**) ROC curves for method 'A', AlphaMissense, and REVEL with the CCV-A test set; (**B**) corresponding PR curves; (**C**) ROC curves for method 'B', AlphaMissense, and REVEL with the CCV-B test set; (**D**) corresponding PR curves. The Ankh language model consistently outperforms ProtT5. However, AlphaMissense and REVEL achieve higher overall predictive performance.

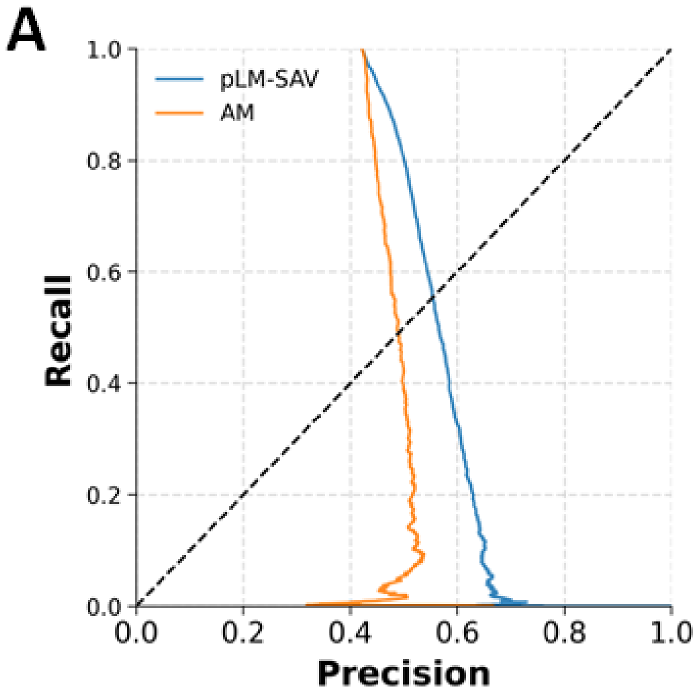

Figure S9. Precision–recall (PR) curves for the pLM-SAV Ankh method ‘B’ on the AMambCV and AMambRCV datasets, as shown in Figure 5 and Table S10. **(A)** pLM-SAV is significantly better than Alphamissesnse; **(B)** REVEL achieves the best performance, while the pLM-SAV model outperforms AlphaMissense.

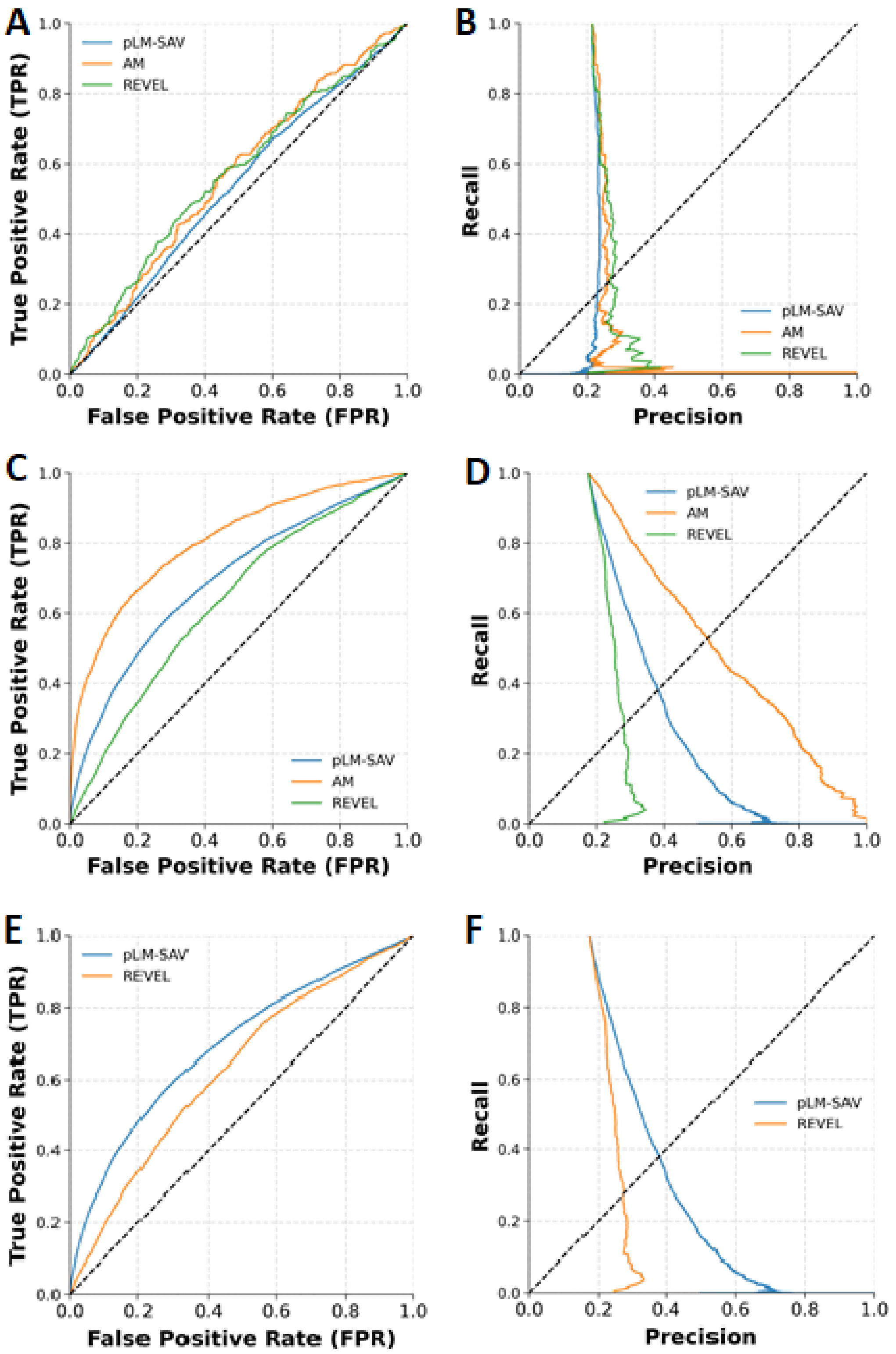

Figure S10. Receiver operating characteristic (ROC) and precision–recall (PR) curves for the pLM-SAV Ankh method 'B' on the remaining ambiguous sets, as shown in Table S10 **(A)** AMambRambCV ROC **(B)** AMambRambCV PR; **(C)** RambAMCV ROC; **(D)** RambAMCV PR; **(E)** RambCV ROC; **(F)** RambCV PR. On the small AMambRambCV set Alphamissense and REVEL are somewhat better than pLM-SAV considering the ROC and PR curves. On the RambAMCV and RambCV sets pLM-SAV outperforms REVEL ambiguous set both in case of ROC and PR, but in case of RambAMCV set AlphaMissense is the best.

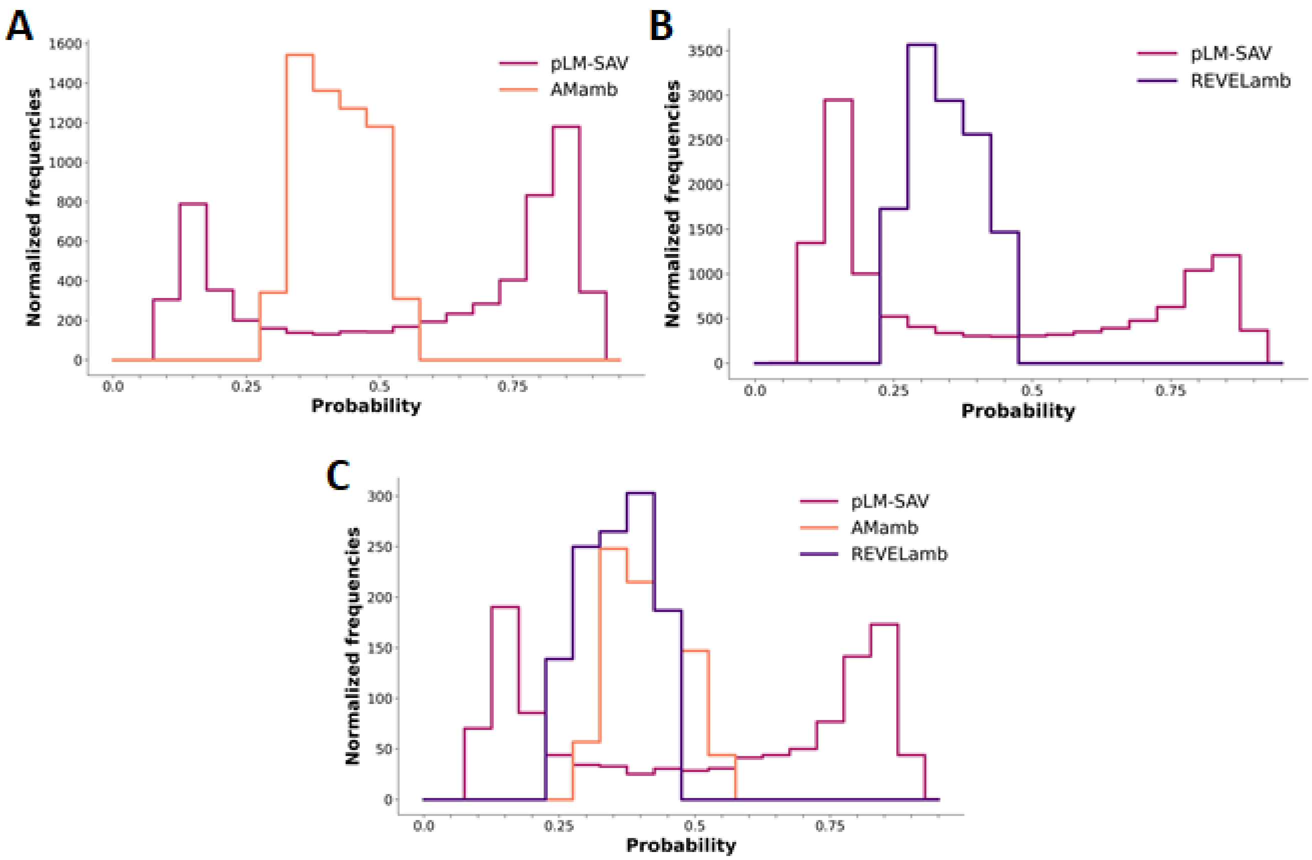

Figure S11. Distribution of pLM-SAV prediction scores in selected ambiguity-defined subsets. Predicted probabilities from the pLM-SAV Ankh method 'B' model are shown for (**A**) AMambCV, (**B**) RambCV, and (**C**) AMambRambCV. AlphaMissense- and REVEL-defined ambiguity does not necessarily correspond to prediction scores near the pLM-SAV decision threshold. These score distributions are descriptive and indicate that pLM-SAV can provide a prediction signal that differs from the uncertainty classifications of AlphaMissense and REVEL.

Table S1. Thresholds for the best pLM-SAV models. For each model, the classification threshold was determined on the validation set by maximizing the Matthews correlation coefficient (MCC). Thresholds from 0.10 to 0.90 were tested in increments of 0.01, and the threshold yielding the highest validation MCC was selected and then applied to the corresponding test set. The best model for each test fold was selected during cross-validation using the criteria AUC > 0.8 and $\min|MCC_{train} - MCC_{valid}|$ and indicated with the validation fold index.

	method 'A' Ankh		method 'A' ProtT5		method 'B' Ankh		method 'B' ProtT5	
test fold index	valid fold index	threshold	valid fold index	threshold	valid fold index	threshold	valid fold index	threshold
0	5	0.52	6	0.45				
1	2	0.38	2	0.37	5	0.55	6	0.57
2	1	0.41	1	0.39	5	0.52	6	0.62
3	1	0.45	1	0.34	5	0.47	6	0.43
4	2	0.42	1	0.43	1	0.66	9	0.53
5	9	0.4	2	0.43	2	0.53	6	0.44
6	3	0.41	7	0.48	1	0.53	5	0.33
7	2	0.37	5	0.27	5	0.41	6	0.51
8	2	0.4	5	0.35	5	0.48	6	0.47
9	3	0.35	5	0.33	1	0.64	2	0.5

Table S2. Performance metrics for the evaluated model architectures. Average AUC and MCC scores (± standard deviation) for the evaluated model architectures on the Eff10k test sets, using the best-performing models of method ‘A’ (10 models from 10-fold cross-validation). The neural network models include dense architectures and convolutional networks. A window size of 7, embeddings from the 7 amino acids were concatenated, resulting in an input feature size of 7×D per variant (D = 1,536 for Ankh and 1,024 for ProtT5). The number of model parameters (N) is also reported.

Some architectures - particularly dense models with window size 7 - contained a very large number of parameters and required up to 63 GiB of memory during training. In contrast, the best-performing model was the compact convolutional architecture CNN1c. Hyperparameter tuning (e.g. learning rate and number of epochs) was performed for this model. Early stopping was applied during training, and in most cases training terminated before reaching 500 epochs, making further increases unnecessary. The best-performing architecture and hyperparameter combination is indicated in bold and highlighted in the main text. For method ‘B’, the same CNN1c architecture was used without additional optimization. A schematic of the CNN1c model is shown in Figure S1, and implementations of all models are available in the *mynn.py* file on Zenodo (https://doi.org/10.5281/zenodo.15502498).

Model	Window size	N parameters	Language model	Epoch	Learning rate	AUC	MCC
NN1	1	7,084,033	Ankh	500	0.0005	0.846 ± 0.0.027	0.573 ± 0.054
NN1	1		ProtT5	500	0.0005	0.841 ± 0.027	0.556 ± 0.049
NN1	7	346,859,521	Ankh	500	0.0005	0.804 ± 0.028	0.575 ± 0.054
NN1	7		ProtT5	500	0.0005	0.802 ± 0.024	0.559 ± 0.047
NN2	1	951,297	Ankh	500	0.0005	0.841 ± 0.0.023	0.572 ± 0.054
NN2	1		ProtT5	500	0.0005	0.840 ± 0.027	0.552 ± 0.047
NN2	7	5,669,889	Ankh	500	0.0005	0.831 ± 0.027	0.570 ± 0.051
NN2	7		ProtT5	500	0.0005	0.835 ± 0.028	0.555 ± 0.050
CNN1c	7	37,193	Ankh	300	0.0005	0.856 ± 0.025	0.571 ± 0.055
CNN1c	7		Ankh	500	0.0001	0.842 ± 0.027	0.0557 ± 0.058
**CNN1c**	**7**		**Ankh**	**500**	**0.0005**	**0.859 ± 0.024**	**0.568 ± 0.049**
CNN1c	7		Ankh	500	0.0008	0.851 ± 0.021	0.565 ± 0.046
**CNN1c**	**7**		**ProtT5**	**500**	**0.0005**	**0.861 ± 0.022**	**0.564 ± 0.047**
CNN2	7	869,001	Ankh	500	0.0005	0.837 ± 0.024	0.566 ± 0.055
CNN2	7		ProtT5	500	0.0005	0.841 ± 0.026	0.559 ± 0.050
CNN3	7	1,435,665	Ankh	500	0.0005	0.830 ± 0.031	0.560 ± 0.056
CNN3	7		ProtT5	500	0.0005	0.773 ± 0.028	0.520 ± 0.048

Table S3. Parameters of the language models used in this study. Both models follow an encoder-decoder architecture. Embeddings were extracted from the encoder output and used for downstream processes, such as training, prediction, and analysis.

model	N(encoder layer)	N(encoder parameters)	Embedding dimensions
Ankh	48	1.15B	1536
ProtT5	24	3B	1024

Table S4. Summary of definitions. The performance of the predictions was evaluated using standard metrics, defined in terms of TP (true positive), FP (false positive), TN (true negative), and FN (false negative).

Metric	Calculation
True positive rate (TPR), recall_effect, sensitivity	TP / (TP+FN)
True negative rate TNR), recall_neutral, specificity	TN / (TN+FP)
$precision_{effect}$	TP / (TP+FP)
$precision_{neutral}$	TN / (TN+FN)
$F1_{effect}$	$2*(precision_{effect}*recall_{effect}) / (precision_{effect}+recall_{effect})$
$F1_{neutral}$	$2*(precision_{neutral} * recall_{neutral}) / (precision_{neutral}+recall_{neutral})$
Accuracy	(TP+TN) / (TP+TN+FP+FN)
MCC	$(TP*TN-FP*FN) / ((TP+FP) * (TP+FN) * (TN+FN) * (TN+FP))^{½}$
AUC (Area Under the Receiver Operating Characteristic Curve)	area under the TPR versus (1 - TNR) curve

Table S5. Quantitative analysis of Δ-embedding windows comparing wild-type and mutant embeddings. Two window sizes were evaluated: w = 1 (mutation position only) and w = 7 (centered on the mutation). The analysis includes N = 99,926 variants from the Eff10k dataset and N = 110,252 ClinVar variants (≥1 star, downloaded November 2023). Δ-embedding vectors were computed as absolute differences, with dimensions (N, D) for w = 1 and (N, 7×D) for w = 7 (D = 1,536 for Ankh and 1,024 for ProtT5). Metrics are grouped according to the level of aggregation:

(i) Per-dimension statistics (blue rows): These metrics summarize variation across variants for each embedding dimension, followed by aggregation across dimensions.

Max(per-dimension max): largest value observed across all embedding dimensions after taking the maximum over variants.

Min(per-dimension max): smallest value observed after taking the maximum over variants.

Max/Min(per-dimension mean) + std: extrema observed after taking the mean over variants (± standard deviation across variants).

These values reflect how strongly individual embedding dimensions capture mutation effects. The corresponding averaged profiles for w = 7 are visualized in Figure S2.

(ii) Global per-dimension average (green row):

Mean and standard deviation computed over all entries of the Δ-embedding matrix, representing the average magnitude of change per embedding dimension.

(iii) Per-variant total difference (brown rows): For each variant, Δ-embedding values were summed across all embedding dimensions, yielding a total difference score per variant (array of size N).

Max/Min:(|Δ-emb| sum): extrema of these total difference scores.

Mean(|Δ-emb| sum) ± std: average total difference across variants with standard deviation.

These values capture the overall embedding shift induced by each mutation across the full window.

Overall, ProtT5 exhibits substantially larger Δ-embedding magnitudes than Ankh across all metrics. However, despite smaller embedding differences, the Ankh model provides superior performance in SAV prediction.

Set	Eff10k		Clinvar	
Embedding window size	1	7	1	7
Language model	Ankh			
Max(per-dimension max)	0.284	0.284	0.322	0.322
Min(per-dimension max)	2.44E-04	5.89E-05	2.76E-04	8.22E-05
Max(per-dimension mean) + std	0.070 ± 0.056	0.070 ± 0.056	0.066 ± 0.058	0.066 ± 0.058
Min(per-dimension mean) + std	2.28E-05 ± 3.78E-05	2.39E-06 ± 5.98E-06	2.34E-05 ± 4.25E-05	1.98E-06 ± 4.03E-06
Mean + std	0.010 ± 0.010	0.004 ± 0.006	0.010 ± 0.009	0.004 ± 0.005
Max(|Δ-emb| sum)	32.777	197.099	37.259	187.185
Min(|Δ-emb| sum)	2.775	8.924	1.592	6.169
Mean(|Δ-emb| sum) + std	15.949 ± 4.334	47.288 ±18.880	14.649 ± 4.109	43.914 ± 13.846
Language model	ProtT5			
Max(per-dimension max)	1.528	1.536	1.705	1.705
Min(per-dimension max)	0.048	0.027	0.064	0.037
Max(per-dimension mean) + std	0.234 ± 0.167	0.234 ± 0.167	0.264 ± 0.165	0.264 ± 0.165
Min(per-dimension mean) + std	0.004 ± 0.005	0.001 ± 0.002	0.004 ± 0.005	0.001 ± 0.002
Mean + std	0.102 ± 0.095	0.040 ± 0.055	0.106 ± 0.101	0.041 ± 0.056
Max(|Δ-emb| sum)	295.193	1426.065	327.539	1656.883
Min(|Δ-emb| sum)	31.490	84.263	28.261	67.685
Mean(|Δ-emb| sum) + std	104.378 ± 32.216	283.523 ± 132.834	108.722 ± 35.148	290.440 ± 119.849

Table S6. pLM-SAV performance on the Eff10k and ClinVar datasets. The Eff10k analysis demonstrates the effectiveness of Δ-embeddings. AUC and MCC values were calculated across Eff10k test folds (Fold *i*) using methods ‘A’ and ‘B’. In addition, pLM-SAV was evaluated on Fold *i* combined with Fold 0 using method ‘B’.

Evaluations were also performed on ClinVar-derived protein sets filtered to avoid sequence-based data leakage. As a result, the ‘A’ (CV-A) and ‘B’ (CV-B) ClinVar evaluations were conducted on partially different datasets, as described in the Methods. To enable a direct comparison between methods ‘A’ and ‘B’, method ‘B’ was additionally applied to the CV-A dataset, which is a subset of CV-B, demonstrating that method ‘B’ outperforms method ‘A’ (last two rows).

Performance was assessed using embeddings from both Ankh and ProtT5 language models. Results are reported as mean ± standard deviation. Statistical significance of differences relative to ‘B’ Ankh Fold i (first value in parenthesis) and ‘B’ Ankh ClinVar CV-B (second value in parenthesis) was evaluated as described in Methods: Performance metrics, corresponding *p*-values are given in parentheses (first compared to ‘a’, second to ‘b’).

Method	AUC	MCC
'A' Ankh Fold i	0.859 ± 0.024 (0.7333349, 0.0000202)	0.568 ± 0.049 (0.4314112, 0.1983787)
'A' ProtT5 Fold i	0.861 ± 0.022 (0.8623825, 0.0000083)	0.564 ± 0.047 (0.3239286, 0.1266627)
'B' Ankh Fold i	0.862 ± 0.018 (a, 0.0000024)	0.585 ± 0.042 (a, 0.6736207)
'B' ProtT5 Fold i	0.844 ± 0.028 (0.1162557, 0.0000046)	0.551 ± 0.048 (0.1270756, 0.0336773)
'B' Ankh Fold i + 0	0.828 ± 0.007 (0.0000111, 0.0000000)	0.501 ± 0.008 (0.0000025, 0.0000000)
'B' ProtT5 Fold i + 0	0.818 ± 0.017 (0.0006748, 0.0003486)	0.478 ± 0.022 (0.0000007, 0.0000000)
'A' Ankh ClinVar CV-A	0.828 ± 0.006 (0.0000301, 0.0000000)	0.298 ± 0.046 (0.0000000, 0.0000000)
'A' ProtT5 ClinVar CV-A	0.784 ± 0.014 (0.0000000, 0.0000000)	0.177 ± 0.024 (0.0000000, 0.0000000)
'B' Ankh ClinVar CV-B	0.906 ± 0.003 (0.0000024, b)	0.592 ± 0.024 (0.6736207, b)
'B' ProtT5 ClinVar CV-B	0.867 ± 0.003 (0.4593151, 0.0000000)	0.450 ± 0.073 (0.0002110, 0.0000475)
'B' Ankh ClinVar CV-A	0.841 ± 0.004 (0.0041855, 0.0000000)	0.378 ± 0.022 (0.0000000, 0.0000000)
'B' ProtT5 ClinVar CV-A	0.804 ± 0.007 (0.0000001, 0.0000000)	0.269 ± 0.056 (0.0000000, 0.0000000)

Table S7. pLM-SAV performance is comparable to supervised methods. Mean MCC and accuracy values and standard errors for PMD4k were collected from published sources, as detailed in the Methods and main text. pLM-SAV Ankh 'A' and 'B' were not statistically different. Differences compared to set 'a' were tested according to the method given in Methods: Performance metrics, *p*-values given in parenthesis.

Method	MCC	Accuracy
Random baseline	-0.002 ± 0.611	0.611 ± 0.41
**Supervised**		
pLM-SAV Ankh 'A'	0.271 ± 0.696 (0.9051662)	0.696 ± 0.038 (0.9065600)
pLM-SAV Ankh 'B'	0.274 ± 0.694 (a)	0.694 ± 0.036 (a)
SNAP2	0.280 ± 0.707	0.707 ± 0.39
VESPA	0.274 ± 0.635	0.635 ± 0.43
VESPAl	0.271 ± 0.63	0.630 ± 0.43
**Rule-based**		
BLOSUM62bin	0.049 ± 0.562	0.562 ± 0.43
ProtT5cons-19equal	0.227 ± 0.686	0.686 ± 0.41
ProtT5-beff	0.16 ± 0.523	0.523 ± 0.43
ConSeq-19equal	0.206 ± 0.715	**0.715 ± 0.39**
ConSeq BLOSUM62	0.138 ± 0.543	0.543 ± 0.43

Table S8. pLM-SAV performance is comparable to rule-based methods. MCC, $F1_{effect}$, $F1_{neutral}$ and accuracy for the human versus non-human split of PMD4k datasets were collected from published sources, as detailed in the Methods and main text. Mean values with standard deviation are shown. pLM-SAV Ankh 'A' and 'B' were statistically different neither in the human nor in the non-human split. Differences compared to set 'a' and 'b' were tested according to the method given in Methods: Performance metrics, *p*-values given in parenthesis.

Method	MCC	$F1_{effect}$	$F1_{neutral}$	Accuracy
**human**				
**pLM-SAV Ankh 'A'**	0.281 ± 0.072 (0.7594396)	0.766 ± 0.038 (0.6242061)	0.492 ± 0.053 (0.8640218)	0.681 ± 0.043 (0.9349253)
**pLM-SAV Ankh 'B'**	**0.292 ± 0.085** (a)	0.763 ± 0.037 (a)	**0.497 ± 0.058** (a)	0.678 ± 0.045 (a)
**SNAP2**	0.24 ± 0.01	0.780 ± 0.6	0.463 ± 1.3	0.688 ± 0.7
**PolyPhen-2**	0.23 ± 0.01	**0.784 ± 0.4**	0.451 ± 1.1	**0.689 ± 0.5**
**SNAP**	0.22 ± 0.01	0.749 ± 0.5	0.467 ± 1.1	0.658 ± 0.6
**SIFT**	0.23 ± 0.01	0.722 ± 0.6	0.490 ± 1.0	0.636 ± 0.6
**non-human**				
**pLM-SAV Ankh 'A'**	0.266 ± 0.062 (0.9615509)	0.789 ± 0.036 (0.8858062)	0.459 ± 0.065 (0.9583687)	0.699 ± 0.040 (0.9037095)
**pLM-SAV Ankh 'B'**	**0.267 ± 0.083** (b)	0.787 ± 0.033 (b)	**0.461 ± 0.077** (b)	0.697 ± 0.039 (b)
**SNAP2**	0.26 ± 0.01	0.799 ± 0.3	0.458 ± 0.8	0.707 ± 0.4
**PolyPhen-2**	0.22 ± 0.01	0.771 ± 0.4	0.447 ± 0.8	0.676 ± 0.5
**SNAP**	0.23 ± 0.01	0.772 ± 0.3	0.455 ± 0.9	0.679 ± 0.5
**SIFT**	0.23 ± 0.01	0.770 ± 0.3	0.458 ± 0.8	0.677 ± 0.4

Table S9. Comparison of the pLM-SAV predictions to AlphaMissense (AM) and REVEL. The CCV-A and CCV-B test sets contained proteins sequentially not similar to those in the training and validation of our pLM-SAV models (method ‘A’ and ‘B’). Metrics were calculated for certain, ambiguous, and all predictions, splitting the data set according to the ambiguous/certain criteria of AlphaMissense or REVEL. Differences compared to set ‘a’…‘j’ were tested according to the method given in Methods: Performance metrics, *p*-values given in parenthesis, line grouping and the same color is applied to indicate the reference set and the *p*-values calculated based on that set. All compared sets were significantly different with $p < 0.05$.

set	method	AUC	MCC	F1effect
CCV-A	'A' Ankh AM ambiguous	0.651 ± 0.009(a)	0.147 ± 0.019(a)	0.258 ± 0.011(a)
	'A' ProtT5 AM ambiguous	0.558 ± 0.013(0.0000000)	0.048 ± 0.022(0.0000000)	0.215 ± 0.007(0.0000000)
	AM ambiguous	0.531 ± 0.038(0.0000000)	0.005 ± 0.042(0.0000000)	0.181 ± 0.031(0.0000000)
	'A' Ankh AM certain	0.857 ± 0.003(b)	0.327 ± 0.051(b)	0.364 ± 0.051(b)
	'A' ProtT5 AM certain	0.822 ± 0.011(0.0000000)	0.199 ± 0.025(0.0000014)	0.256 ± 0.018(0.0000065)
	AM certain	0.957 ± 0.003(0.0000000)	0.687 ± 0.011(0.0000000)	0.711 ± 0.010(0.0000001)
	'A' Ankh REVEL ambiguous	0.677 ± 0.006(c)	0.191 ± 0.036(c)	0.431 ± 0.017(c)
	'A' ProtT5 REVEL ambiguous	0.611 ± 0.007(0.0000000)	0.083 ± 0.020(0.0000001)	0.386 ± 0.007(0.0000004)
	REVEL ambiguous	0.635 ± 0.020(0.0000000*)	0.157 ± 0.032(0.0009616)	0.398 ± 0.024(0.0000156)
	'A' Ankh REVEL certain	0.865 ± 0.003(d)	0.320 ± 0.049(d)	0.343 ± 0.051(d)
	'A' ProtT5 REVEL certain	0.836 ± 0.011(0.0000001)	0.198 ± 0.025(0.0000016)	0.237 ± 0.018(0.0002122)
	REVEL certain	0.961 ± 0.003(0.0000000)	0.741 ± 0.011(0.0000000)	0.763 ± 0.010(0.0000000)
	'A' Ankh all	0.848 ± 0.003(e)	0.315 ± 0.047(e)	0.355 ± 0.047(e)
	'A' ProtT5 all	0.809 ± 0.011(0.0000000)	0.192 ± 0.024(0.0000008)	0.253 ± 0.017(0.0002122)
	AM all	0.950 ± 0.003(0.0000000)	0.628 ± 0.010(0.0000000)	0.657 ± 0.010(0.0000000)
	REVEL all	0.950 ± 0.003(0.0000000)	0.648 ± 0.011(0.0000000)	0.682 ± 0.010(0.0000000)
CCV-B	'B' Ankh AM ambiguous	0.668 ± 0.006(f)	0.207 ± 0.021(f)	0.506 ± 0.009(f)
	'B' ProtT5 AM ambiguous	0.631 ± 0.006(0.0000000)	0.128 ± 0.038(0.0000621)	0.471 ± 0.003(0.0000000)
	AM ambiguous	0.594 ± 0.011(0.0000000)	0.132 ± 0.019(0.0000000)	0.441 ± 0.013(0.0000002)
	'B' Ankh AM certain	0.918 ± 0.002(g)	0.615 ± 0.024(g)	0.729 ± 0.017(g)
	'B' ProtT5 AM certain	0.885 ± 0.004(0.0000000)	0.473 ± 0.078(0.0000862)	0.634 ± 0.053(0.0001026)
	AM certain	0.963 ± 0.001(0.0000000)	0.810 ± 0.003(0.0000000)	0.865 ± 0.002(0.0000000)
	'B' Ankh REVEL ambiguous	0.692 ± 0.005(h)	0.216 ± 0.019(h)	0.376 ± 0.013(h)
	'B' ProtT5 REVEL ambiguous	0.643 ± 0.005(0.0000000)	0.135 ± 0.022(0.0000003)	0.326 ± 0.009(0.0000001)
	REVEL ambiguous	0.632 ± 0.009(0.0000000)	0.146 ± 0.013(0.0000000)	0.335 ± 0.010(0.0000000)
	'B' Ankh REVEL certain	0.923 ± 0.002(i)	0.634 ± 0.023(i)	0.749 ± 0.016(i)
	'B' ProtT5 REVEL certain	0.890 ± 0.004(0.0003486)	0.490 ± 0.079(0.0000817)	0.656 ± 0.052(0.0000892)
	REVEL certain	0.972 ± 0.001(0.0000000)	0.813 ± 0.003(0.0000002)	0.869 ± 0.002(0.0000002)
	'B' Ankh all	0.908 ± 0.002(j)	0.590 ± 0.024(j)	0.713 ± 0.016(j)
	'B' ProtT5 all	0.873 ± 0.004(0.0003486)	0.454 ± 0.075(0.0000810)	0.624 ± 0.050(0.0000967)
	AM all	0.957 ± 0.001(0.0000000)	0.767 ± 0.003(0.0000000)	0.835 ± 0.002(0.0000000)
	REVEL all	0.963 ± 0.001(0.0000000)	0.739 ± 0.003(0.0000000)	0.814 ± 0.002(0.0000000)

Table S10. AUC and MCC scores of pLM-SAV, AlphaMissense, and REVEL predictions for different ambiguous subsets with ClinVar label. The AMambCV set is the largest possible variant set that included ambiguous AlphaMissense with ClinVar labels; its subset is the AMambRCV set, where the variants have any kind of REVEL predictions. Similarly the RambCV set is the largest possible variant set that included ambiguous REVEL with ClinVar labels and its subset, the RambAMCV set contains variants with any kind of AM predictions. AMambRambCV contains ambiguous Alphamissense and ambiguous REVEL variants with ClinVar labels and it is the intersection of AMambRCV and RambAMCV. In case of the AMambCV, RambCV, AMambRCV and RambAMCV sets pLM-SAV gave significantly better predictions than the method with ambiguous variants, although in case of AMambRCV REVEL gave the best predictions and in case of the RambAMCV set AM proved to be the best. On the smallest AMambRambCV set both AM and REVEL gave a bit better predictions than pLM-SAV, although for AM MCC the difference was not significant. The variants comprising the AMambRambCV set might have some problematic features regarding the predictions as both AM and REVEL struggled to categorize them sufficiently, regardless the different approaches used. These variants are not necessarily 'ambiguous' (meaning close to the thresholds of the pLM-SAV models) as it can be seen on Fig. S10, but still can be difficult to predict well with pLM-SAV as well. Differences compared to set 'a'-'e' were tested according to the method given in Methods: Performance metrics, *p*-values given in parenthesis, line grouping and the same color is applied to indicate the reference set and the *p*-values calculated based on that set. All compared sets were significantly different with $p < 0.05$ except the AMambRambCV MCC score.

set	method	AUC	MCC
AMambCV	'B' Ankh	0.664 ± 0.009$^{(a)}$	0.234 ± 0.025$^{(a)}$
	AM	0.576 ± 0.007$^{(0.0000000)}$	0.118 ± 0.013$^{(0.0000000)}$
AMambRCV	'B' Ankh	0.660 ± 0.009$^{(b)}$	0.224 ± 0.024$^{(b)}$
	AM	0.576 ± 0.008$^{(0.0000000)}$	0.116 ± 0.014$^{(0.0000000)}$
	REVEL	0.842 ± 0.005$^{(0.0000000)}$	0.445 ± 0.010$^{(0.0000000)}$
AMambRambCV	'B' Ankh	0.534 ± 0.016$^{(c)}$	0.051 ± 0.031$^{(c)}$
	AM	0.566 ± 0.021$^{(0.0000054)}$	0.070 ± 0.030$^{(0.0523404)}$
	REVEL	0.568 ± 0.021$^{(0.0000015)}$	0.077 ± 0.029$^{(0.0054839)}$
RambAMCV	'B' Ankh	0.697 ± 0.008$^{(d)}$	0.227 ± 0.019$^{(d)}$
	AM	0.806 ± 0.006$^{(0.0000000)}$	0.415 ± 0.010$^{(0.0000000)}$
	REVEL	0.633 ± 0.007$^{(0.0000000)}$	0.150 ± 0.009$^{(0.0000000)}$
RambCV	'B' Ankh	0.696 ± 0.009$^{(e)}$	0.224 ± 0.018$^{(e)}$
	REVEL	0.626 ± 0.007$^{(0.0000000)}$	0.143 ± 0.009$^{(0.0000000)}$

Table S11. pLM-SAV prediction speed compared to VESPAl. The only available benchmark data for VESPA was for its lightweight version, VESPAl. Under this approximate comparison, the pLM-SAV 'A' models achieve similar prediction speeds to VESPAl, while the 'B' models are more than twice as fast. The pLM-SAV speed tests were performed on an NVIDIA GeForce RTX 2080 Ti. The VESPAl benchmark reported by its authors was performed on an NVIDIA Quadro RTX 8000.

method	N(models)	N(variants)	N(predictions)	time (s)	prediction/s
VESPAl	10	380,000	3,800,000	2,400	1,583
'A' Ankh	10	17,677	176,770	130	1,360
'A' ProtT5	10	17,677	176,770	125	1,414
'B' Ankh	9	83,642	752,778	180	4,182
'B' ProtT5	9	83,642	752,778	165	4,562

Table S12. Effect of secondary mutations on pLM-SAV predictions. We evaluated whether additional mutations at secondary sites influence the predicted pathogenicity of a primary mutation using the 'B' Ankh model. For CFTR (UniProt: P13569), the ΔF508 deletion was approximated by the F508G substitution, as pLM-SAV does not support deletions. The F508G mutation was predicted to be pathogenic (probability: 0.800) and this prediction remained unchanged even in the presence of two different sets of known rescue mutations (3PT and 6SS without ΔRI) (Soya, N. et al. 2023). For human LILRB4 (UniProt: Q8NHJ6), we examined the mutation of Cys144 – part of a disulfide bridge in the Ig-like domain – which was predicted to be pathogenic. We hypothesized that a compensatory mutation at the corresponding cysteine (C195) might rescue the prediction. Several combinations were tested, including substitutions to charged residues (D) and small residues (A, G), but none resulted in a reduction in predicted pathogenicity. All these results suggest that such secondary mutations do not systematically influence the model's output.

protein	mutation	secondary site mutation(s)	probability
CFTR	F508G	none	0.800
	F508G	S492P, A534P, I539T	0.803
	F508G	M470V, S492P, S495P, A534P, I539T, R555K	0.803
LILRB4	C144K	none	0.913
	C144K	C195D	0.908
	C144A	none	0.904
	C144A	C195A	0.879
	C144G	none	0.904
	C144G	C195A	0.897

References

Cheng J, Novati G, Pan J *et al.* Accurate proteome-wide missense variant effect prediction with AlphaMissense. *Science* 2023;**381**:eadg7492.

Notin P, Dias M, Frazer J *et al*. Tranception: protein fitness prediction with autoregressive transformers and inference-time retrieval. In: Chaudhuri K, Jegelka S, Song L et al., eds. Proc 39th Int Conf Mach Learn. PMLR 2022;162:16990–17017.

Soya N, Xu H, Roldan A *et al.* Folding correctors can restore CFTR posttranslational folding landscape by allosteric domain–domain coupling. *Nat. Commun.* 2023;**14:**6868.

Paszke A, Gross S, Massa F *et al.* PyTorch: An Imperative Style, High-Performance Deep Learning Library. In: Wallach H, Larochelle H, Beygelzimer A, et al., eds. *Advances in Neural Information Processing Systems*. Vol 32. Curran Associates, Inc., 2019.
